# Neutrophil Recruitment to Lymph Nodes Limits Local Humoral Response to *Staphylococcus aureus*

**DOI:** 10.1371/journal.ppat.1004827

**Published:** 2015-04-17

**Authors:** Olena Kamenyeva, Cedric Boularan, Juraj Kabat, Gordon Y. C. Cheung, Claudia Cicala, Anthony J. Yeh, June L. Chan, Saravanan Periasamy, Michael Otto, John H. Kehrl

**Affiliations:** 1 B-Cell Molecular Immunology Section, Laboratory of Immunoregulation, National Institute of Allergy and Infectious Diseases, National Institutes of Health, Bethesda, Maryland, United States of America; 2 Biological Imaging Section, Research Technologies Branch, National Institute of Allergy and Infectious Diseases, National Institutes of Health, Bethesda, Maryland, United States of America; 3 Pathogen Molecular Genetics Section, Laboratory of Human Bacterial Pathogenesis, National Institute of Allergy and Infectious Diseases, National Institutes of Health, Bethesda, Maryland, United States of America; 4 Immunopathogenesis Section, Laboratory of Immunoregulation, National Institute of Allergy and Infectious Diseases, National Institutes of Health, Bethesda, Maryland, United States of America; Trinity College Dublin, IRELAND

## Abstract

Neutrophils form the first line of host defense against bacterial pathogens. They are rapidly mobilized to sites of infection where they help marshal host defenses and remove bacteria by phagocytosis. While splenic neutrophils promote marginal zone B cell antibody production in response to administered T cell independent antigens, whether neutrophils shape humoral immunity in other lymphoid organs is controversial. Here we investigate the neutrophil influx following the local injection of *Staphylococcus aureus* adjacent to the inguinal lymph node and determine neutrophil impact on the lymph node humoral response. Using intravital microscopy we show that local immunization or infection recruits neutrophils from the blood to lymph nodes in waves. The second wave occurs temporally with neutrophils mobilized from the bone marrow. Within lymph nodes neutrophils infiltrate the medulla and interfollicular areas, but avoid crossing follicle borders. *In vivo* neutrophils form transient and long-lived interactions with B cells and plasma cells, and their depletion augments production of antigen-specific IgG and IgM in the lymph node. *In vitro* activated neutrophils establish synapse- and nanotube-like interactions with B cells and reduce B cell IgM production in a TGF- β1 dependent manner. Our data reveal that neutrophils mobilized from the bone marrow in response to a local bacterial challenge dampen the early humoral response in the lymph node.

## Author Summary

Highly antibiotic resistant *Staphylococcus aureus* (*S*. *aureus*) are an important human pathogen and major cause of hospital acquired infections. An early host defense mechanism against bacterial infection is neutrophil recruitment, which helps eliminate the bacteria at the site of invasion. However, unless quickly neutralized, pathogens such as *S*. *aureus* can gain access to nearby lymph nodes via draining lymphatics. Lymph nodes protect the host by mobilizing additional resources that limit further pathogen dissemination. These include recruitment of neutrophils to the lymph node to directly target pathogens and the initiation of adaptive immune mechanisms, such as the humoral immune response, which transforms B lymphocytes capable of making pathogen specific antibodies into antibody producing plasma cells. Using a mouse model that allows direct visualization of lymphocytes, neutrophils, and fluorescently-labeled *S*. *aureus* in lymph nodes, we document the rapid appearance of bacteria in the lymph node following local *S*. *aureus* infection. We characterize the dynamic influx of neutrophils that occurs as a consequence and reveal direct B cell-neutrophil interactions within the lymph node parenchyma. We find that while lymph node neutrophils rapidly engage bacteria, they limit the subsequent humoral immune response likely by producing Transforming Growth Factor-β1, a factor known to limit B cell responses. These finding have important implication for our understanding of B cell responses against potent pathogens such as *S*. *aureus* and for the design of effective vaccines.

## Introduction

Lymph nodes (LNs) are secondary lymphoid organs where pathogenic antigens are captured and processed, and antigen-specific (adaptive) responses are generated. T and B cells arrive to the LNs with the blood flow or via the afferent lymphatics, and occupy highly specialized compartments (niches) to differentiate into effector cells [1, 2]. At the same time, LN residing innate cells shape these adaptive response directly by capturing antigens and either eliminating or presenting them, and indirectly by creating cytokine-rich surroundings [3]. Among the latter, neutrophils are the most dynamic cells mobilized to the LNs following infection or immunization [4, 5]. While activated neutrophils are known for their capability to either support lymphocyte proliferation and activation [6] or suppress adaptive cell function [7], the physiological roles of their influx to the LNs following vaccination or during the course of an infection remain only partially understood.

Mature neutrophils express Ly6G^hi^, CXCR2, and CXCR4; and reside in the bone marrow (BM) niche retained by high concentration of SDF-1α [8], and in the red pulp of the spleen [9]. During inflammation neutrophils are mobilized to the blood and migrate toward the source of CXC chemokines and other mediators released by affected cells or pathogens [10] to liquidate the source of danger [11]. Concurrently, they infiltrate adjacent lymphoid tissues to perform other highly specialized tasks, often linking innate and adaptive immunity [12]. In challenged LNs, neutrophils support cell-mediated responses during the differentiation of T_h_1 and T_h_17 cells, and development of efficient T_h_2 mediated response [13, 14]. However, suppressive effect of neutrophils on T cell mediated response have also been shown [15, 16]. Neutrophils augment antibody production by facilitating marginal zone B cell responses in spleen [17], and can favor the transition from autoimmunity to lymphoma [18]. Conversely, depletion of neutrophils in mice immunized with protein antigens in adjuvants leads to elevated levels of serum antibodies [19].

The formation of a productive humoral response in LNs depends upon proper B cell trafficking and highly orchestrated intercellular interactions. After B cells exit high endothelial venules (HEVs), they migrate through the medullary region (MR) and interfollicular zones (IFZ) to populate follicular areas near the subcapsular sinus (SCS) [20]. Follicular B cells exposed to cognate antigen migrate to the follicle border to acquire T cell help, and either proceed to the IFZ to differentiate into early antibody secreting cells or re-enter follicles to form germinal centers (GCs). GC B cells clonally expand and differentiate into plasma cells (PCs) or memory B cells [21]. Terminal B cell differentiation is accompanied by increasing expression of the transcription factor BLIMP-1 [22], and often takes place within the IFZ, and along the medullary cords. PCs predominately reside in the MR, or leave the LN to localize in splenic red-pulp or in specialized BM niches [23]. B cell proliferation and maturation can be boosted by cytokines like BAFF, APRIL and IL-6 released by innate cells [24], or inhibited in T cell contact-depended manner [25] or by cytokines like TGF- β 1 [26]. Sites or niches where recruited neutrophils reside in LN and their regulatory effects on LN B cells are largely unknown.

*Staphylococcus aureus* (*S*. *aureus*) is a potent human pathogen and the most common cause of skin and soft tissue infections in the USA. The host mobilizes both innate and adaptive immune responses to counter the infection. While neutrophils provide an initial line of defense arriving rapidly at the invasion site, the importance of humoral immunity in pathogen clearance is unresolved [27, 28]. Some studies dispute its importance emphasizing the role of cellular immunity and in particular the importance of T_h_1 and T_h_17 cells [29]. Supporting this view B cell deficiency does not worsen the level of *S*. *aureus* bacteremia [30]. Yet multiple bacterial virulence factors specifically target humoral immunity [31]. For example, the humoral immune response is suppressed by *S*. *aureus* superantigens, which activate antimicrobial B cell populations triggering activation-induced cell death [32] and *S*. *aureus* protective antigens suppress B cell response [33]. LAC is a clone of methicillin-resistant *S*. *aureus* (MRSA) strain USA300 (known as Los-Angeles County clone) that compromises severely both innate and adaptive immunity of the host [34]. Detailed understanding the mechanisms of neutrophil and B cell responses to LAC is an urgent need in order to develop an effective anti-Staphylococcal vaccine strategy [35]. In this study we asked how the massive neutrophil recruitment that occurs during local *S*. *aureus* infection might impact the humoral immune response in the draining LN.

We analyzed the mobilization of neutrophils to the inguinal LN (iLN) challenged with heat-inactivated or live *S*. *aureus* using intravital two-photon laser scanning microscopy (TP-LSM). Our *in vivo* data indicate that the migration areas of mobilized neutrophils and activated B cells in the iLN often overlapped, while neutrophils and B cells established multiple intercellular interactions enriched with F-actin. The early humoral response to *S*. *aureus* in the iLN was significantly boosted after neutrophil depletion *in vivo*, and BLIMP1^+^ GC B cell numbers were elevated. Shown *in vitro*, activated neutrophils secreted TGF-β1, which potently suppressed IgM production by iLN B cells. To specify the origin of neutrophils recruited to the iLN, we performed intravital microscopy of mouse calvarium and demonstrated neutrophil egress from the BM prior to their mobilization to the iLN. Our results suggest that the mobilization of bone marrow neutrophils to LNs following immunization or infection acts to limit the early humoral response.

## Results

### Neutrophils enter the iLN via HEVs to infiltrate the MR and IFZ, but avoid LN follicles

**Fig 1:**
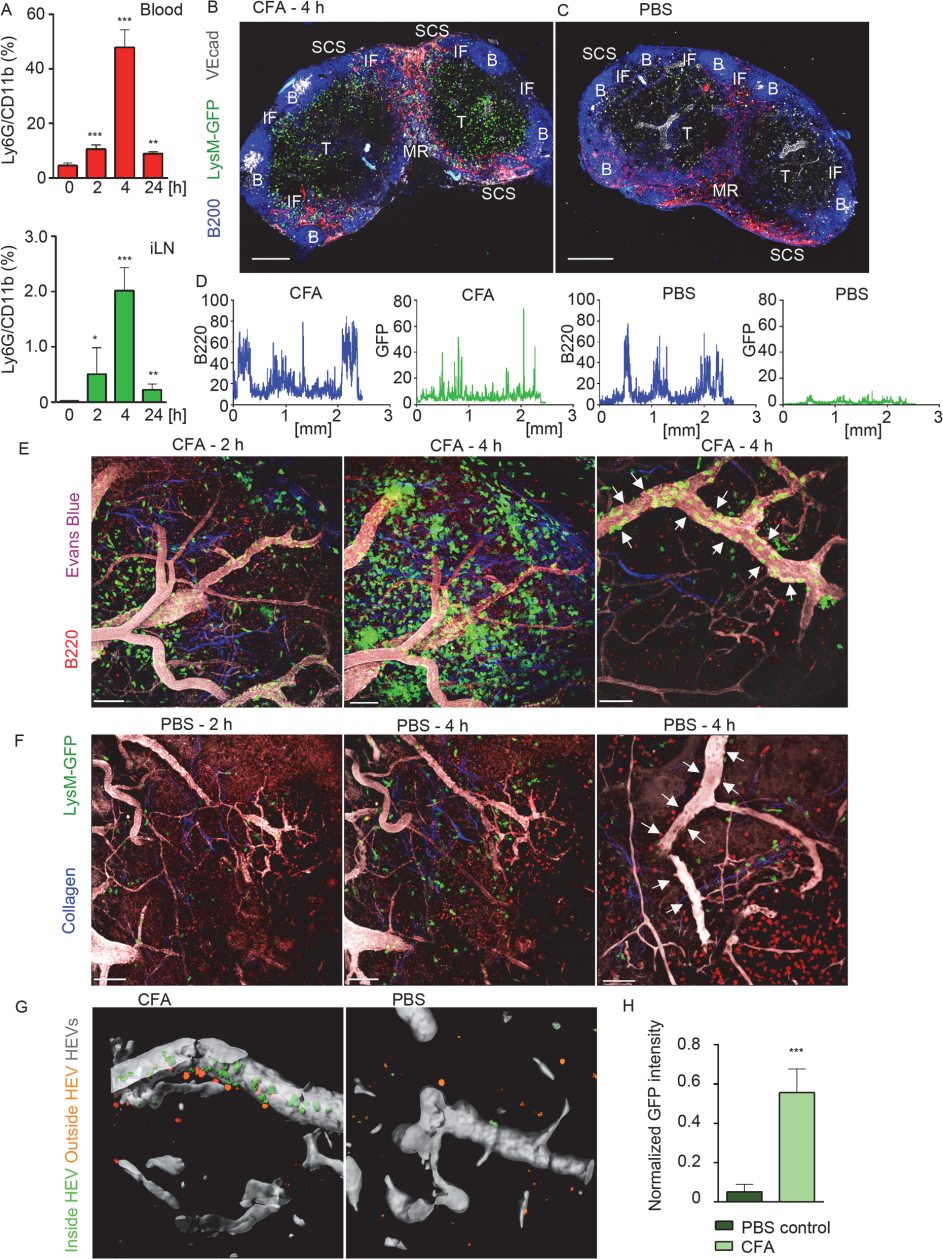
Neutrophils arrive from HEVs to occupy IFZ, MR and SCS in immunized iLN. Mice were injected subcutaneously with CFA. C57BL/6 mice were subjected to flow cytometry analysis of whole blood and LN cell populations; LysM-GFP expression was imaged using confocal microscopy or TP-LSM. (**A**) Flow cytometry analysis of blood (upper panel) and iLN cells (lower panel) in immunized C57BL/6 mice between 0 and 24 h after injection. Percentages of Ly6G^hi^/CD11b^hi^ population in live cell gate are shown. N = 2 mice/4 iLNs, repeated 3 times. Means ± SEM (**B, C**) Mice were sacrificed 4 h after CFA or PBS injections. The iLNs were sectioned, immunostained and analyzed by confocal microscopy. Single Z stack images were collected and assembled to form a large tiled image of the whole iLN. TZ (T), IFZ (IF), medulla (MR), LN follicle (B), and SCS are labeled. Tiled confocal images of (B) immunized and (C) PBS injected control LysM-GFP iLNs with GFP^hi^ neutrophils (green), B cells (B220, blue), lymphatics (LYVE-1, red) and blood vessels (VE-cadherin, gray) are shown. Scale bars: 300 μm; Z = 35 μm. (**D**) GFP (green) and B220 (blue) channels were split, profiles of intensities of fluorescence were plotted across the images of immunized and control iLNs and measured on a scale from 1 to 100. X axis: distance in mm. Representative for 10 random profiles plotted across each section. The images are representative of 10 mice analyzed. (**E, F**) For TP-LSM B cells (CMTPX, red) were adoptively transferred 24 h prior to imaging; blood vessels were visualized via intravenous injection of EB (gray); collagen fibers were seen as second harmonic generation (blue). TP-LSM images of (E) immunized (S1 Movie) and (F) PBS control iLN at 2 and 4 h after injections. Scale bars: 70 μm (left and middle panels). Single HEVs (white arrows) at 4 h after injections are shown. Scale bars: 50 μm (right panels). (**G**) An HEV volume was defined using Imaris, and neutrophils were distinguished as cells inside (green) or outside the blood vessel (orange) in immunized (left) and PBS control (right) iLN. (**H**) GFP intensity of cells inside HEVs was calculated for 5 random blood vessels in immunized versus PBS control iLN, and normalized for a blood vessel volume. 5 repeats; means ± SEM.

A previous study had shown neutrophil recruitment to the iLN following the local injection of Complete Freunds’s adjuvant **(**CFA) [36]. CFA is composed of inactivated and dead *M*. *tuberculosis* emulsified in mineral oil. It is commonly used to enhance humoral immunity and is part of some induction schemes for triggering autoimmunity in mice. To provide a basis for comparison to *S*. *aureu*s injected mice, we assessed local neutrophil response following subcutaneous CFA injection near the iLN (S1A Fig). Analysis of cell mobilization kinetics indicated a peak of neutrophil recruitment approximately 4 h after CFA immunization both in the blood and in the iLN that subsided nearly to base line the following day (Fig 1A). Ly6G^+^/CD11b^+^ cell population increased 10 fold in the blood (S1B Fig) and 8 fold in the iLNs (S1C and S1D Fig). Both the percentage and overall number of B220^+^ cells also increased in the LN by 24 h after CFA injection, while CD4^+^ and CD8^+^ T cells numbers remained unchanged (S1E Fig).

Confocal microscopy was used to examine live LN sections from LysM-GFP mice (MGI:2654931, S1 Table), injected with CFA or PBS. LysM is highly expressed in neutrophils and at lower levels in other myeloid cells; therefore, neutrophils can be distinguished on the basis of their morphology and strong GFP expression [37]. Our analysis showed that GFP^hi^ cells concentrated within the SCS, MR, T cell zone (TZ), and IFZ in CFA immunized iLN at 4 h post-injection (Fig 1B). In contrast, the PBS injected mouse had only a rare GFP^hi^ cell (Fig 1C). A comparison of GFP fluorescence intensities (neutrophils) indicated the presence of multiple cells in immunized and only few in control iLN, while B220 fluorescent intensities (B cells) were analogous at this time point (Fig 1D). *In vivo*, GFP^hi^ cells were mobilized to the iLN 2 h after CFA administration, rapidly increasing their numbers, thereafter (Fig 1E and S1 Movie). Neutrophils arrived initially via the SCS and blood vessels; however, they entered iLN parenchyma predominantly by exiting blood vessels. Inside the capillaries, GFP^hi^ cells displayed signs of early leukocyte diapedesis: rolling, adhesion, and arrest (S1 Movie). ILN in PBS-injected control contained only a rare neutrophil after 2 h, no further infiltration was observed, and the microvasculature was free of neutrophils (Fig 1F, arrows). Analysis of normalized mean GFP fluorescence within the HEVs confirmed abundant presence of GFP^hi^ cells only in immunized iLN (Fig 1G and 1H).

To determine whether neutrophils could be recruited to B cell follicles, we induced laser damage within a follicle. Between 0 and 1 h neutrophils exited the HEVs near the follicle and migrated directly to injury site forming a swarm (S2 Movie and S1F and S1G Fig). 1.5 h later neutrophils left the follicle, perhaps via chemorepulsion [38], as many recently swarmed cells moved backwards partially clearing the area. These data show that mobilized neutrophils infiltrate the SCS, MR, and IFZ of the iLN. After 2 h of CFA challenge, neutrophils infiltrate the iLN parenchyma arriving from the blood microcirculation. Neutrophils avoid entering iLN follicles; however, a local injury can trigger their immediate entry.

### Neutrophils swarm and interact with lymphocytes in iLN of *S*. *aureus* immunized mice

**Fig 2:**
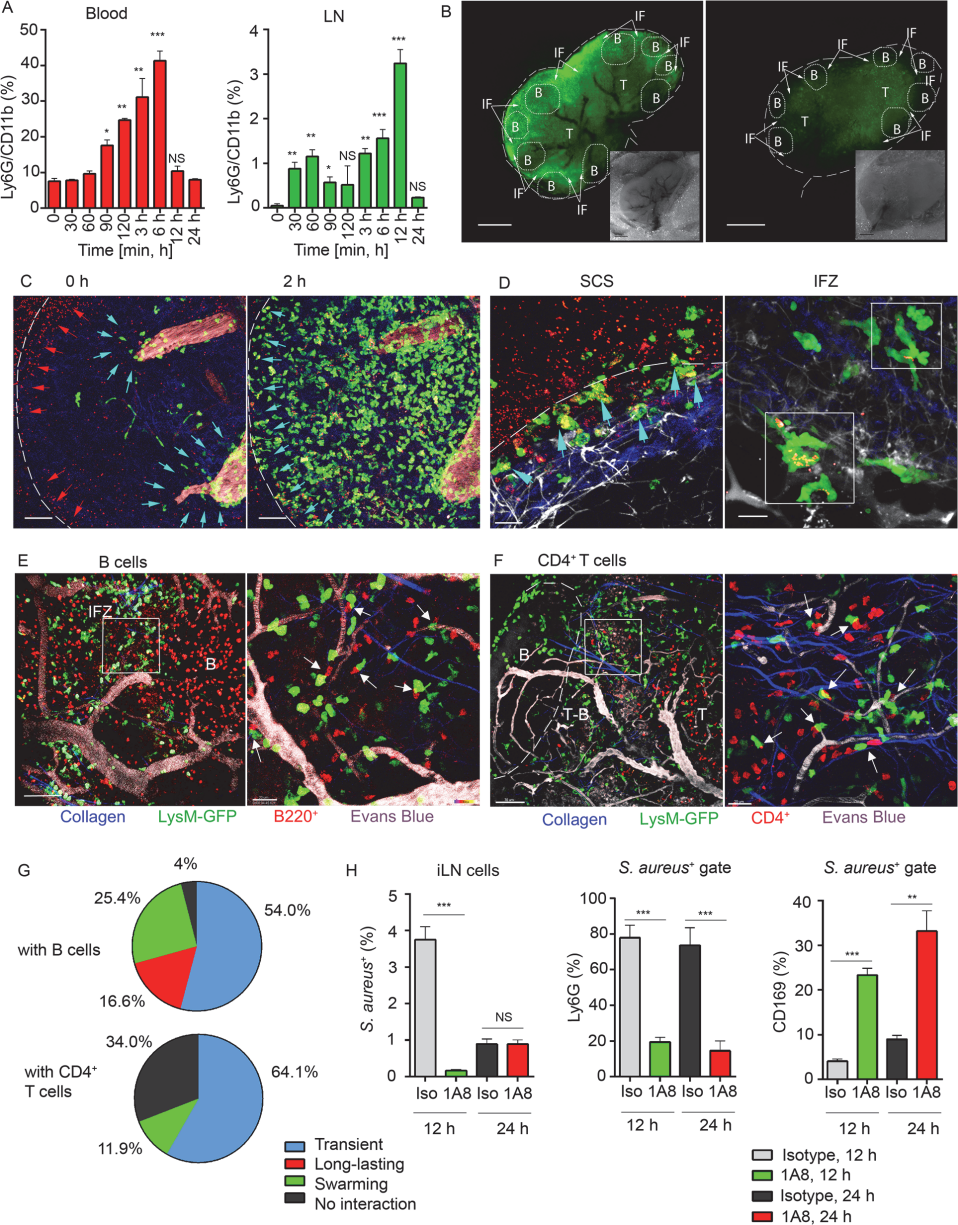
Local immunization with *S*. *aureus* bioparticles recruits neutrophils to the LN. *S*. *aureus* bioparticles were opsonized and injected subcutaneously near the iLN. Neutrophil recruitment to the blood and lymphoid organs was analyzed by flow cytometry in C57BL/6 mice, or imaged using epifluorescent stereomicroscope or TP-LSM in LysM-GFP mice with adoptively transferred lymphocytes. (**A**) Kinetics of neutrophil recruitment to the blood (left panel) and to the iLN (right panel) between 0 and 24 h after immunization. N = 3 mice/6 iLNs; 3 independent experiments. Means ± SEM. (**B**) Fluorescent (main image) and bright field (lower right corner) images of immunized and PBS control LysM-GFP LNs are shown at 12 h after injection. Neutrophils: LysM-GFP, green. Image labeling: SCS (white dashed line), TZ (T), IFZ (IF, white arrows), LN follicles (B, dotted lines). Data is representative of 3 mice (6 iLNs) per group. (**C**) TP-LSM images of neutrophils (LysM-GFP, green) exiting blood vessels in the LN stroma (left panel, blue arrowheads) and accumulating in the SCS (right panel, blue arrowheads) between 2 h (left) and 4 h (right) after *S*. *aureus* (red arrowheads) injection are shown (S3 Movie, Mobilization). ILN border, white dashed line; HEVs, EB, gray. Scale bars: 50 μm. (**D**) Neutrophils phagocytizing *S*. *aureus* in the SCS (left panel, blue arrowheads) and swarming in the IFZ (right panel, white squares) between 3 and 4 h after *S*. *aureus* inoculation are shown (S3 Movie, Swarming). Scale bars: 25 μm (right), 20 μm (left). Data is representative of 12 imaging sessions. (**E, F**) DsRed B cells or CD4^+^ T cells were adoptively transferred 24 h prior to imaging. Neutrophil (green) interactions with (E) B cells (red) or (F) CD4^+^ T cells (red) at the T-B border (dashed line) at 12 h after *S*. *aureus* injection are shown. Blood vessels, EB (gray); collagen, second harmonic (blue). Scale bars: (C) 50 μm; (D, left) 50 μm; (D, right) 20 μm, (E, left) 50 μm; (E, right) 20 μm; (F, left) 70 μm; (F, right) 20 μm. (**G**) The percentages of short and long-lasting interactions formed by neutrophils with B cells (upper chart) and by neutrophils with CD4^+^ T cells (lower chart). Data is representative of 3 independent computations. (**H**) Analysis of *S*. *aureus* uptake by Ly6G^+^ and CD169^+^ populations in the iLN of isotype control or 1A8-injected mice between 0 and 48 h after immunization. N = 4 iLNs; 3 repeats. Means ± SEM.

Next, we studied neutrophil influx to the iLN in response to a local injection of inactivated *S*. *aureus* Wood 46 strain. Analysis of recruitment kinetics was expanded to time points between 0 and 120 min, and at 2, 3, 6, 12 and 24 h. The neutrophils increased in the blood 1 h post-injection, continued to increase reaching a plateau at 6 h, and returned to baseline by 12 h (Fig 2A, left). Over the same interval we detected two waves of neutrophils infiltrating the iLN, 1^st^ between 0 and 60 min with the peak at 30 min, and 2^nd^ between 2 and 24 h with a plateau between 6 and 12 h. Their percentage had returned almost to baseline by 24 h (Fig 2A, right). Epifluorescent microscopy of intact LysM-GFP iLN showed abundant presence of GFP^hi^ cells in the SCS (white dashed line) and IFZ (IF, white arrows) of immunized iLN at 12 h (Fig 2B). We also analyzed recruitment of neutrophils to distant LNs choosing the axillary and superficial cervical LNs and to the spleen at 4 and 12 h after immunization. Along with the massive influx of neutrophils to the iLN, we detected a significant recruitment to the spleen, but none to distant LNs (S2A and S2B Fig).

Shown by intravital TP-LSM of the iLN in immunized LysM-GFP mice, *S*. *aureus* bioparticles arrived with the lymph flow (S3 Movie, Mobilization, red arrowheads) and partially accumulated in the SCS (Fig 2C, left panel, red arrowheads). The influx of neutrophils to the iLN gradually increased between 2 and 4 h (Fig 2C, right panel; S3 Movie, Mobilization, green). Mobilized from the blood stream (Fig 2C, blue arrowheads), they first infiltrated the MR and the IFZ, then between 2 and 3 h migrated from the parenchymal regions to the SCS, where they phagocytized bacterial particles (Fig 2D, left panel, blue arrowheads). Between 3 and 6 h, neutrophils loaded with bioparticles moved back into the iLN parenchyma and swarmed in the MR and IFZ (S3 Movie, Swarming, white square). The swarms usually accumulated bioparticles trapped by the neutrophils (Fig 2D, right panel, while squares).

By 12 h after bioparticle injection, while B cells migrated within the follicle and in the IFZ, many of neutrophils were recruited to the follicle border (S4 Movie). At the follicle border, they formed associations with B cells, mostly in the perivascular regions (Fig 2E, left). In more detail, after exiting HEVs neutrophils encountered B cells that migrated or oscillated along the outer vessel wall. When a B cell appeared in close proximity to a neutrophil, the cells often clustered (Fig 2E, right). Remarkably, while most of neutrophils only transiently interacted with B cells, others formed persistent cell-cell contacts (S5 Movie). In transient interactions, neutrophils usually formed protrusions, wrapped around lymphocytes (S2C Fig), and then left, while B cells responded by attempting to follow the departing neutrophils. Such interactions usually resolved within 10 to 60 sec (S5 Movie, arrows). The persistent interactions typically involved arrested neutrophils that formed tight intercellular contacts with B cells lasting 30 min and longer (S2D Fig and S5 Movie, white circles). We also found formation of multiple cell-cell contacts between recruited neutrophils and CD4^+^ T cells migrating within TZ and reaching the T-B border (Fig 2F). Quantitative analysis of cell-cell interactions showed both short and long-lasting interactions between neutrophils and B cells (Fig 2G, upper chart), while all interactions of GFP^hi^ cells with CD4^+^ cells were transient (Fig 2G, lower chart; S6 Movie, arrows). Only GFP^lo^ cells (DCs) formed long-term interactions with CD4^+^ T cells (S6 Movie, blue circles).

We also analyzed LN cell populations that predominantly engulfed *S*. *aureus*. Approximately 4% of the total LN cells were *S*. *aureus* positive at 12 h after injection (S2E Fig) and 1% after 24 h. As expected, more than 80% of the positive cells were neutrophils, while the other positive cells included CD169^+^ and CD169^-^ macrophages along with CD11c^+^ DCs (S2F Fig). As neutrophils provided the bulk of the clearance, we asked which cell type would clear *S*. *aureus* in their absence. For this, we depleted mice of neutrophils followed by bioparticle injection. While in isotype control mice 5–10% of the CD169^+^ macrophages contained bioparticles, in the depleted mice, this percentage increased to 25–35% (Fig 2H). These results indicate that the local injection of *S*. *aureus* induces a rapid recruitment of neutrophils to the adjacent LNs and spleen. Mobilized LN neutrophils swarm and intense neutrophil phagocytosis ensues. Multiple neutrophil interactions with B cells and CD4^+^ T cells occur. In the absence of neutrophils, LN CD169^+^ macrophages more actively participate in bacterial particle clearance.

### *S*. *aureus* infection induces continuous recruitment of neutrophils to adjacent LNs followed by neutrophil phagocytosis

**Fig 3:**
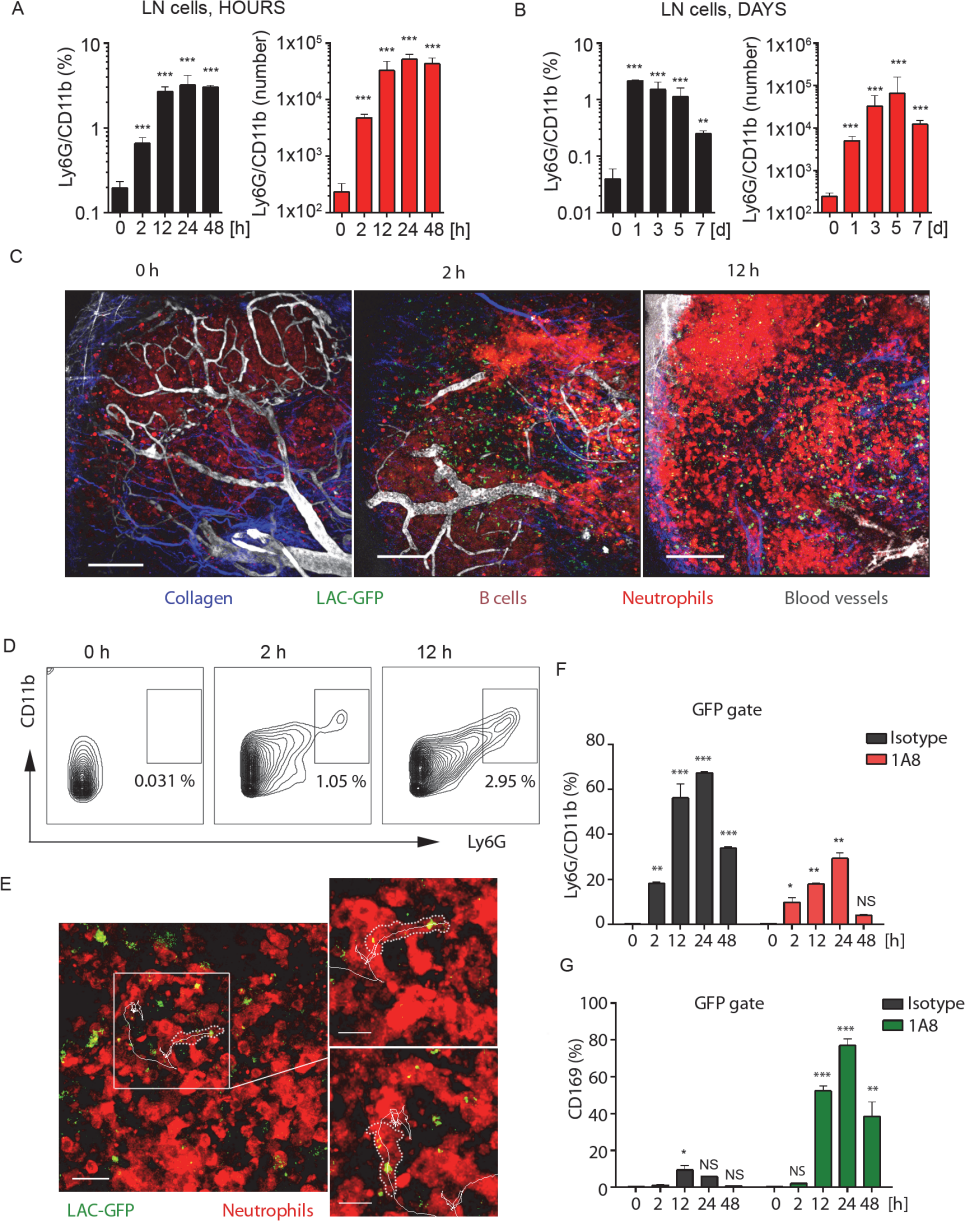
Local LAC-GFP infection recruits neutrophils to the iLN. C57BL/6 or dsRed BM chimeric mice were injected subcutaneously near the iLN with LAC-GFP in amount of 1 x 10^5^ CFU per iLN or given PBS as a control. Neutrophil recruitment to the iLN was analyzed by flow cytometry between 0 and 48 h. PBS injected control is shown as a time point 0 of infection. DsRed chimeric mice were imaged using TP-LSM between 2 and 12 h after infection. (**A**) Kinetics of neutrophil mobilization to the iLN between 0 and 48 h after infection is shown. (**B**) Kinetics of neutrophil mobilization to the iLN between days 0 and 7 after infection is shown. (A, B) Percentages of Ly6G^hi^/CD11b^hi^ population (left) and total Ly6G^hi^/CD11b^hi^ cell numbers per iLN (right) in live cell gate are shown. N = 4 mice/8 iLNs. Means ± SD. (**C**) TP-LSM images of neutrophil accumulation (dsRed^hi^, bright red) in iLN at 0 h (left), 2 h (middle), and 12 h (right) after LAC-GFP (green) injection are shown (S7 Movie). Blood vessels (EB, gray); collagen (second harmonic, blue). IFZ (IF), LN follicle (B, dotted line), and LN borders (dashed line) are labeled. Scale bars: 50 μm. (**D**) At indicated time points, representative flow cytometry plots show Ly6G^hi^/CD11b^hi^ cell population in the iLN of infected dsRed mice. Data is representative of 4 iLNs. (**E**) 2 min migration route of a single neutrophil (red) loaded with LAC-GFP bacteria (green) is shown. IFZ, 6 h after infection (S8 Movie). Enlarged images of the same cell at time points 1 and 100 sec are shown to the right. Neutrophil cell border is outlined with dotted line; cell track is shown with solid line. Scale bars: 20 μm (left), 5 μm (right). Data is representative of 4 imaging sessions. (**F**, **G**) Infected and control mice were injected with isotype control or 1A8 antibody at 100 μg/mouse on day -1 and 0 of infection and LN cells were analyzed for GFP signal using flow cytometry. Analysis of LAC-GFP uptake by (F) Ly6G^hi^/CD11b^hi^ and (G) CD169^+^ populations in the iLN of isotype control or 1A8 injected mice between 0 and 48 h after infection is shown. N = 4 mice/8 iLNs. Means ± SD.

Next, we studied neutrophil recruitment to the iLN after local *S*. *aureus* infection. LAC-GFP derivative of USA300 was used as a live *S*. *aureus* strain. Consistent with earlier observations in CFA and *S*. *aureus* bioparticle immunized mice, local LAC-GFP infection caused rapid and massive influx of neutrophils to the iLN (Fig 3A and 3B). Analysis of mobilization kinetics, however, revealed more abundant (total Ly6G^+^/CD11b^+^ cell number per iLN) and continuous (percentage over time) neutrophil influx after the infection comparing to immunization (Figs 2A, 3A, and S2A). While in infected mice the peak of recruitment was observed by 12 h after the infection, neutrophil numbers did not drop by 24 h (Fig 3A). Neutrophil influx to the iLN following infection continued as their numbers were elevated until at least day 7 post-infection (Fig 3B).

To visualize early events of neutrophil recruitment to the iLN after local LAC-GFP infection, we performed TP-LSM using dsRed (MGI:3663358, S1 Table) bone marrow chimeras. In these mice all hematopoietic cells express dsRed; however, in infected mice mobilized neutrophils were distinguished by their high intensity of fluorescence (S3A and S3B Fig). Flow cytometry analysis of LAC-infected mice at 24 h after infection (S3 Fig) showed that neutrophils mobilized to the blood stream (S3A Fig) and to the iLN (S3B Fig) were dsRed^hi^, and B cells dsRed^med/lo^ (S3B Fig, lower left panel). ILN of PBS injected dsRed bone marrow chimeric mice was free of dsRed^hi^ cells (S3B Fig, lower-right panel). Additionally, neutrophils in LAC-GFP infected iLN were identified due to their distinct morphology and behavior, i.e. size, dynamic migration, swarming and phagocytosis of the bacteria. Imaging neutrophil influx to the iLN between 2 and 12 h after LAC-GFP injection revealed their rapid mobilization from the blood vessels to the MR, TZ, IFZ and eventually to the SCS (Fig 3C and S7 Movie). At indicated time points, Ly6G^+^/CD11b^+^ cell population in the iLN of infected dsRed mice increased from 0.3 to 3–5% (Fig 3D). Abundant presence of neutrophils was also observed in the iLN at 24 h after the infection using TP-LSM (S3C Fig). Mobilized neutrophils infiltrated the iLN parenchyma where they intensively swarmed and phagocytized LAC-GFP. Many of recruited neutrophils carried GFP^+^ bacteria while migrating and swarming in the iLN (Fig 3E, white square; S8 Movie, white circles and cell tracks). While not as potent as the *S*. *aureus*, local instillation of sheep red blood cells (SRBC) near the iLN or injection of a standard protein antigen in alum (NP-KLH) also recruited neutrophils to the LN, while their recruitment was slower. Neutrophils were found in SRBC-immunized iLN by day 3 after immunization, and localized mostly in IFZ and at B cell follicle borders (S3D Fig, arrowheads).

We also examined uptake of LAC-GFP by SCS macrophages in infected iLN after neutrophil depletion (Fig 3F and 3G). In isotype control mice the majority of LAC-GFP^+^ cells were neutrophils (Fig 3F). Consistent with previously observed in *S*. *aureus* bioparticle-immunized mice, in LAC-GFP infected mice depleted of neutrophils, the CD169^+^ macrophage population that contained LAC-GFP (CD169^+^ within GFP gate of live LN cells) was increased to 75–80% (Fig 3G). This percentage was elevated comparing to previously observed during immunization (Fig 2H). This data shows rapid and continuous influx of neutrophils to the iLN adjacent to LAC-GFP infection site. While recruited neutrophils rapidly phagocytize the majority of LAC in the iLN, in absence of neutrophils SCS macrophages uptake the bacteria.

### Neutrophils and B cells establish tight and nanotube-like intercellular contacts enriched with F-actin

**Fig 4:**
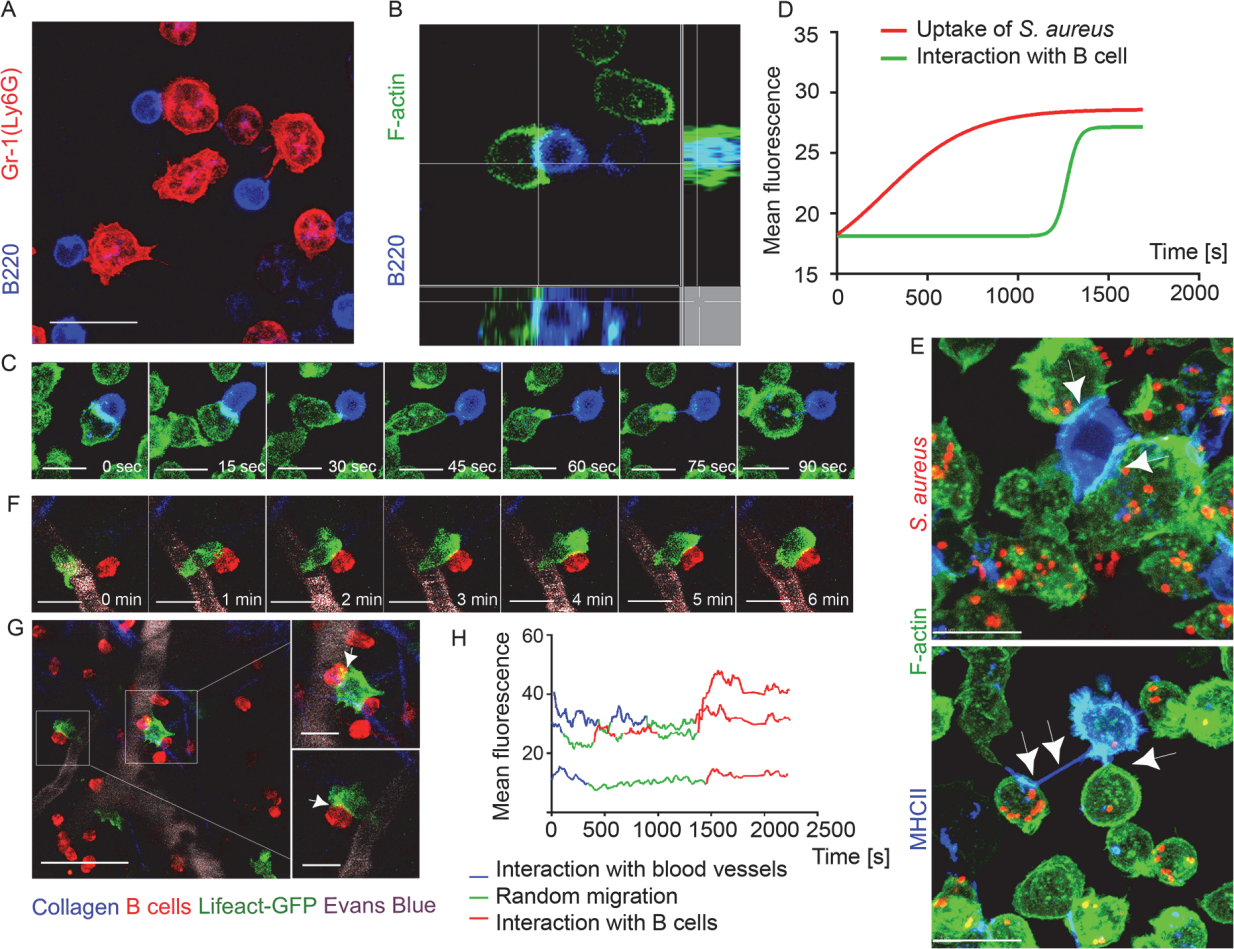
F-actin accumulates at interaction sites between neutrophils and B cells. Lifeact-GFP neutrophils and B cells were co-cultured on ICAM-1+VCAM-1+KC coated surface and imaged using confocal microscopy. Lifeact-GFP neutrophils and dsRed B cells were adoptively transferred into C57BL/6 mice 24 h prior to imaging. Mice were injected subcutaneously with *S*. *aureus* bioparticles near the iLNs, and imaged using TP-LSM 12 h after immunization. (**A**) A live-cell confocal image of BM derived neutrophils (Ly6G, red) and LN derived B cells (B220, blue) co-cultured for 2 h. Scale bar: 10 μm. (**B**) Single-plane confocal image of F-actin at a cell-cell contact between a neutrophil (Lifeact-GFP, green) and a B cell (B220, blue). (**C**) Time-lapse series of images shows F-actin clustering at the leading edge of a neutrophil interacting with a B cell. Scale bars: 7 μm. (**D**) Comparison of F-actin assembly during *S*. *aureus* phagocytosis by neutrophils and during neutrophil-B cell interactions measured as increase in the GFP fluorescence intensity (S9 Movie). 25 cells analyzed, curves averaged and fitted using GraphPad Prism software. (**E**) Live-cell confocal images of F-actin enriched tight cell-cell contacts (upper image, arrowheads) and nanotubes (lower image, arrowheads) formed between neutrophils (Lifeact-GFP, green) and B cells (MHCII, blue) co-cultured for 2 h, and with *S*. *aureus* bioparticles added to the co-cultures. Scale bars: 7 μm. (**F**) Time-lapse series of TP-LSM images showing steps of short-term interaction between a Lifeact-GFP neutrophil (green) and a B cell (red) in perivascular space *in vivo*. Scale bars: 7 μm. (**G**) Lifeact-GFP neutrophils (green) encountering B cells (red) near blood vessels (EB, gray); scale bar: 25 μm. Enlarged images (squares to the right); scale bars: 10 μm. (**H**) F-actin clustering in neutrophils during their egress from the blood vessels, random migration, and interaction with B-cells measured as GFP mean fluorescence intensity. The fluorescence fluctuation in 3 cells is shown; 25 cells analyzed.

To investigate neutrophil-B cell interactions observed in immunized mice, we imaged neutrophils and B cells from Lifeact-GFP mice (MGI:4831036, S1 Table). In these mice, filamentous actin (F-actin) can be visualized due to GFP expression during F-actin assembly [39]. Lifeact-GFP mice were immunized locally near the iLN with *S*. *aureus* bioparticle, and isolated Ly6G^hi^ cells and B220^+^/MHCII^+^ B cells were studied both *in vitro* and *in vivo*. The Ly6G^hi^ cells formed prominent cellular protrusions that contacted B220^+^/MHCII^+^ B cells, after both cell types adhered to ICAM-1/VCAM-1/KC coated plates (Fig 4A). Live cell time-lapse confocal microscopy revealed that the intercellular contacts were enriched with F-actin (Fig 4B), and B cell-neutrophil interactions induced rapid clustering of F-actin at the leading edge of neutrophils (Fig 4C). When bioparticles were added to the co-cultures, Lifeact-GFP neutrophils rapidly acquired F-actin during bioparticle uptake (S4A–S4C Fig and S9 Movie) as well as during formation of cell-cell interactions (S4D–S4F Fig and S9 Movie). However, the quantitative analysis of GFP mean fluorescence revealed that F-actin accumulated more rapidly in neutrophils engaging B cells than during bioparticle phagocytosis (Figs 4D, S4C and S4F). B cells in return formed tight membrane associations with neutrophils (Fig 4E, upper panel) and fine membrane protrusions or nanotubes (Fig 4E, lower panel).

We also imaged F-actin enriched B cell-neutrophil intercellular contacts *in vivo*. Shown using TP-LSM in mice with adoptively transferred dsRed B cells and Lifeact-GFP neutrophils, F-actin formation initially occurred at neutrophil leading edge and later at cell-cell contact sites (Fig 4F). The majority of observed interactions occurred when both cell types were arrested in perivascular space near the blood vessels (Fig 4G). Quantification of F-actin assembly in Lifeact-GFP neutrophils measured as increase in GFP mean fluorescence showed increases during formation of intercellular contacts, equal or higher to that detected in the same neutrophils detaching from blood vessels post-diapedesis (Fig 4H). These experiments show direct synapse-like and nanotube-like interaction between neutrophils and B cells in immunized mice. These intercellular contacts are enriched with F-actin that accumulates at a cell-cell contact area within seconds.

### Depletion of neutrophils boosts antibody production by LN B cells, while activated neutrophils suppress antibody production via TGF-β1 production

**Fig 5:**
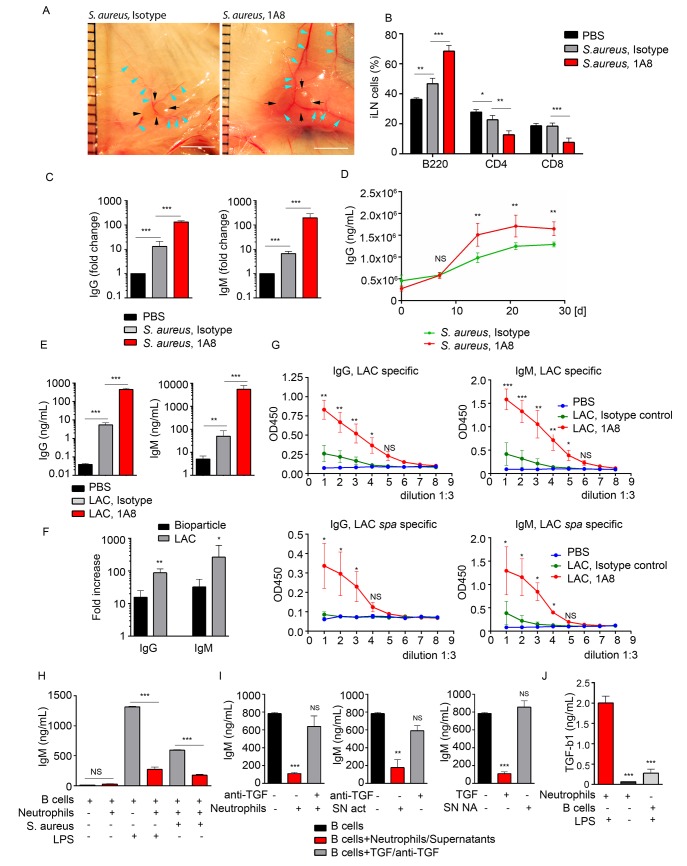
Neutrophils limit the humoral response following local immunization or infection. C57BL/6 mice were given PBS as a baseline control, immunized with *S*. *aureus* bioparticles, or infected with LAC-GFP. Isotype control or 1A8 antibody was injected intraperitoneally at 100 μg/mouse on day -1, 0 and 1 of immunization/infection. (**A**) Images of iLNs in isotype control (left) and neutrophil depleted (right) mice immunized with *S*. *aureus* bioparticles are shown at day 5 after immunization. Indicated are LN edges (black arrows), blood vessels (blue arrows). Scale bars: 5 mm. (**B**) Flow cytometry analysis of B and T cell populations in the iLN of isotype control or neutrophil-depleted mice 3 days after immunization. N = 4 iLNs; 3 repeats. Means ± SEM. (**C**) ELISA of total IgG and IgM produced by iLN B cells isolated from PBS control, immunized isotype control and immunized neutrophil-depleted (1A8) mice. B cells were isolated from the iLNs at day 6 after immunization and cultured for 3 days. N = 4 iLNs. Data are shown as fold change. 3 repeats. Means ± SEM. (**D**) ELISA of total IgG in the serum of immunized isotype control and immunized neutrophil-depleted mice measured at days 0, 7, 14, 21 and 28 after immunization. N = 4 mice; 2 repeats; means ± SEM. (**E-G**) Mice were depleted of neutrophils as above and infected with LAC-GFP. (**E**) ELISA of total IgG and IgM produced by iLN B cells. ILNs were harvested at day 5 after infection, B cells were isolated and cultured for 3 days. N = 5–7 iLNs. Data are shown as antibody concentration in B cell supernatants. Means ± SD. (**F**) Comparison of fold changes in IgG and IgM production in neutrophil-depleted and isotype iLNs, calculated for *S*. *aureus* immunized versus LAC-GFP infected mice. Data shown as fold increases (Means ± SD). (**G**) ELISA of LAC-specific (upper panel) and LAC *spa*-specific IgG and IgM. N = 5–7 iLNs. Means ± SD. (**H**) ELISA of IgM produced *in vitro* by LPS or *S*. *aureus* activated LN B cells in presence of neutrophils, activated correspondingly. No LPS in culture used as a baseline control. (**I**) ELISA of IgM produced by LPS activated iLN B cells in presence of LPS-activated neutrophils supernatants from LPS-activated (SN act) and non-activated neutrophils (SN NA), neutralizing anti-TGF-β1 antibody, and TGF-β1. (**J**) ELISA results measuring TGF-β1 produced by activated neutrophils, non-activated neutrophils or LPS-activated B cells in 24 h cultures. (H-J) Final concentration of LPS in all B cell cultures: 2 μg/mL. 3 to 5 repeats for each of *in vitro* experiments. Means ± SEM.

The large influx of neutrophils and their observed interactions with B cells following local injection of *S*. *aureus* suggested that these interactions might influence the subsequent humoral response. To test this possibility we depleted neutrophils *in vivo* and measured antibody production by iLN B cells in mice immunized with *S*. *aureus* bioparticles or infected with LAC-GFP. The mice received an intraperitoneal injection of Ly6G-specific antibodies (1A8) or isotype control antibodies at day -1, 0 and 1 of immunization/infection with *S*. *aureus*. 24 h after first 1A8 injection, neutrophils were mobilized to the blood and LNs in *S*. *aureus* immunized isotype control-injected, but not 1A8-injected mice (S5A–S5C Fig). At day 5, the iLNs in neutrophil-depleted mice were larger, and more heavily vascularized than in isotype control mice (Fig 5A). Analysis of the kinetics of lymphocyte recruitment to *S*. *aureus* bioparticle-immunized iLN revealed an increase in B220^+^ cell population and decrease in CD4^+^ and CD8^+^ populations in neutrophil-depleted mice (Fig 5B). B220^+^ cell numbers increased in neutrophil-depleted mice correlating with the total iLN cell numbers (S5D Fig). We harvested the iLN B cells at days 5–6 post *S*. *aureus* injection, cultured them for 3 days and measured the levels of IgG and IgM in the supernatants. We compared amounts of antibodies produced by B cells derived from a single iLN (S5D Fig). In the LNs from mice injected with *S*. *aureus* bioparticles, neutrophil depletion caused a 12-fold increase in total IgG and 30-fold increase in total IgM production (Figs 5C and S5E). When quantified as amount of antibodies per B cell number, antibody production was also increased in B cell cultures derived from neutrophil-depleted mice (S5F Fig). Total IgG levels were elevated in the serum of neutrophil-depleted mice starting at day 14 after immunization with *S*. *aureus* bioparticles (Fig 5D). In the LNs harvested from LAC-GFP infected mice, neutrophil depletion resulted in over a 100-fold increases in both IgG and IgM production by LN B cells (Fig 5E). Thus, the fold increase in antibody production after neutrophil depletion was higher in LAC-GFP infected mice than in the *S*. *aureus* bioparticle immunized mice (Fig 5F). Using LAC or LAC *spa* lysates as antigens, we found that LAC-specific IgG and IgM responses were elevated in neutrophil-depleted mice (Fig 5G). At day 5 after infection, LAC was found in the LNs of neutrophil depleted mice but not of isotype control-injected mice (S3G Fig).

To determine if neutrophil depletion also augmented LN B cells responses to protein antigens we isolated LN B cells 7 days after immunization and measured their secretion of IgG and IgM. In case of SRBCs we measured total IgG and IgM production and for the NP-KLH immunized mice we measured NP-KLH specific IgG and IgM produced by LN B cells. In both instances neutrophil depletion resulted in a higher production of antibody (S5H and S5I Fig).

To provide insight into the mechanism by which activated neutrophils suppress LN B cell antibody production we established an *in vitro* system. We isolated B cells from the iLNs of naïve mice and activated them with either LPS or *S*. *aureus* in the presence of absence of neutrophils. In the co-culture we chose a ratio of 10 B cells to 1 neutrophil as that is the approximate ratio of B cells to neutrophils in the immunized iLN. We relied on the ability of LPS or *S*. *aureus* to activate both B cell antibody production and to stimulate neutrophils. We found that both inductive signals increased IgM production in the B cell cultures. When neutrophils were present we observed a potent suppression of IgM production (Figs 5H and S5J, left). At the same time, we did not observe such a pronounced reduction of IgA levels in LN B cell cultures in presence of *S*. *aureus* bioparticle-activated neutrophils (S5J Fig, right). Seeded at the same cell density, by day 5 B cell numbers in B cell/neutrophil co-cultures were 1.5-fold lower than in pure B cell cultures (S5K Fig). Thus, in presence of activated neutrophils, IgM production by total LN B cell cultures was 5-fold suppressed and IgA production 2-fold suppressed (S5L Fig, left). When normalized for B cell number (production by 1 x 10^6^ B cells), IgM production was still 4-fold decreased, and IgA production only 35% decreased (S5L Fig, right).

Next, we tried to identify the inhibitor present in the activated neutrophil cultures. As TGF-β1 is known as a potent inhibitor of B cell antibody production [26, 40], we added a neutralizing TGF-β1 antibody to B cell-neutrophil co-culture. The suppressive effect of neutrophils was nearly completely reversed (Fig 5I, left). In addition, supernatant from LPS-activated neutrophils (SN act), but not from non-stimulated cells (SN non-act), also suppressed IgM production, and this effect was reversed by adding a neutralizing TGF-β1 antibody (Fig 5I, middle and right). We also verified that LPS-activated neutrophils secrete TGF-β1, much more than non-activated neutrophils or LPS-activated B cells (Fig 5J). These data indicate that neutrophils mobilized to antigen stimulated LNs can suppress B cell antibody production and suggest that this may occur via neutrophil TGF-β1 production. An increase in humoral immune response in neutrophil-depleted mice infected with live *S*. *aureus* is more pronounced than in those immunized with *S*. *aureus* bioparticles, SRBC or NP-KLH.

### BLIMP1-YFP^+^ B cell population is increased in neutrophil-depleted mice

**Fig 6:**
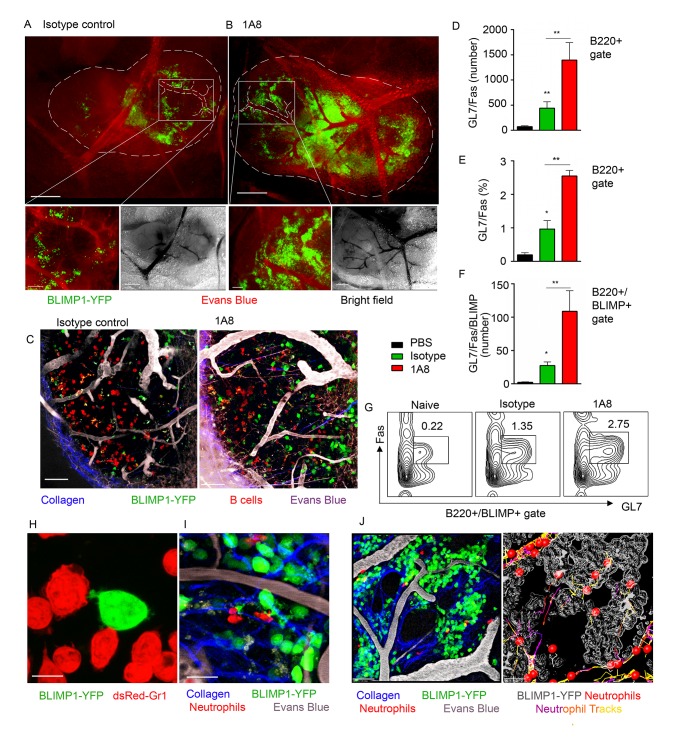
Neutrophil depletion results in increased population of BLIMP1-YFP+ and GC B cells in immunized iLN. BLIMP1-YFP BM reconstituted mice were immunized with *S*. *aureus* subcutaneously, and injected with isotype control antibody or depleted of neutrophils using 1A8 antibody at days -1, 0 and 1 of immunization. ILNs were examined using epifluorescent microscopy, TP-LSM or flow cytometry. (**A, B**) Immunized mice were injected intravenously with Evans blue, and the iLNs of euthanized mice were exposed on a skin flip for imaging using stereomicroscope. Epifluorescent images of intact iLNs in immunized mice injected with (A) isotype control or (B) 1A8 antibody are shown. BLIMP1-YFP^+^ cells (green); blood vessels (red). Fluorescent images (top); corresponding bright field images (bottom left); scale bars, 400 μm. Lymph node borders and single blood vessels in IFZ are shown with dashed lines. Representative perivascular areas occupied with BLIMP1-YFP^+^ cells within the IFZ are outlined with white squares and their enlarged images are shown (bottom right); scale bars: 100 μm. (**C**) DsRed B cells or BM derived neutrophils were adoptively transferred at day 1–2 after immunization. TP-LSM images show LN follicles (red) from an immunized iLN of an isotype control-injected (left) or neutrophil-depleted (right) mouse. Scale bars, 20 μm. (**D-F**) Results from flow cytometry analysis of GC B cell (D) number and (E) percentage within the B220^+^ gate; and (F) BLIMP1-YFP^+^ GC B cell number within the B220^+^ gate. (**G**) Representative flow cytometry patterns of GC B cells from isotype control or 1A8 treated mice. (D-G) N = 4 iLNs; 3 independent experiments. Means ± SEM (**H**) BLIMP1-YFP B cells were isolated from the iLN and activated with LPS *in vitro* for 72 h. DsRed neutrophils were isolated from the BM and activated with LPS *in vitro* for 24 h. The cells were co-cultured for 2 h on ICAM-1/VCAM-1 coated surface. A confocal image of neutrophils (dsRed, red) interacting with a B cell (YFP, green) is shown. Scale bar: 5 μm. (**I**) DsRed BM derived neutrophils were adoptively transferred at day 7 after immunization, and recipient mice imaged 24 h later. A TP-LSM image of neutrophils (dsRed, red) interacting with B cells (YFP, green) is shown. Blood vessels (EB, gray). Scale bar: 10 μm. (**J**) Representative TP-LSM image of BLIMP1-YFP^+^ cells (green) localized in perivascular niches (S1 Movie) with neutrophils (red) migrating through the niches (left panel). Neutrophil tracks localized within the inside perimeter (outline, gray) of the niches (right panel). Scale bar: 20 μm. (H-J) Representative of 3 experiments.

To analyze the impact of neutrophil influx on generation of early PC population in the LN we utilized mice expressing a BLIMP1-YFP transgene (MGI: 99655, S1 Table). BLIMP1-YFP mice were injected with isotype control or 1A8 antibodies and immunized with *S*. *aureus* bioparticles near the iLN. Consistent with previous characterization of PC development in BLIMP1-YFP mice [41], we identified YFP^hi^ cells in the BM and the iLN of *S*. *aureus* immunized mice at day 7, but not at day 3 after immunization. As shown using epifluorescent stereomicroscopy at day 8 after immunization, YPF^hi^ cells localized mostly in the perivascular regions in the MR and IFZ in the iLN of isotype control-injected mice (Fig 6A, white square). In the iLN of neutrophil-depleted mice, YPF^hi^ cells were more abundant in the MR and IFZ, and more tightly packed around the blood vessels within these regions (Fig 6B, white square). Furthermore, YFP^med^ cells found in B cell follicles were more numerous in the neutrophil-depleted mice (Fig 6C). As shown by flow cytometry analysis the neutrophil-depleted mice had more B220^+^ cells (S6A Fig) and more GL7^+^Fas^+^ cells within B220^+^ gate per iLN than isotype control mice (Fig 6D and 6E). 2–6% of the B220^+^GL7^+^Fas^+^ cells were also BLIMP1-YFP^+^ (Fig 6F). These cells were enriched in the neutrophil-depleted mice and are likely the same cells observed in LN follicles using TP-LSM (Fig 6C). A typical flow cytometry pattern of the B220^+^ gated cells analyzed for GL7 and FAS expression from control mice and depleted mice is shown (Fig 6G).

*In vitro*, LPS activated BLIMP1-YFP^+^ cells established cell-cell contacts with BM derived neutrophils by forming both tight interactions and membrane arms (Fig 6H). Intercellular interactions between neutrophils and BLIMP1-YFP^+^ cells were also present in *S*. *aureus* immunized iLN with dsRed BM derived neutrophils adoptively transferred 12 h prior to imaging (Fig 6I). Flow cytometry analysis of LNs in mice immunized with *S*. *aureus* bioparticles confirmed that population of Ly6G^+^/CD11b^+^ cells was increased at day 7 after immunization (S6B and S6C Fig). More than 80% of this population represented endogenous neutrophils versus those adoptively transferred prior to imaging (S6D Fig). Shown by TP-LSM *in vivo*, BLIMP1-YFP^+^ cells occupied distinctive perivascular niches, and neutrophils accumulated within the perimeter of these niches (Fig 6J). While some of mobilized neutrophils formed short cell-cell contacts with BLIMP1-YFP^+^ cells along their migratory tracks, others were arrested inside the niches in clusters with BLIMP1-YFP^+^ cells (S10 Movie). Imaging live sections of immunized BLIMP1-YFP^+^ iLN at day 7 after *S*. *aureus* bioparticle injection has shown similar localization of Ly6G^hi^ cells to that observed during initial neutrophil recruitment: in the MR, IFZ and TZ, often clustered around blood vessels (S6E Fig, arrows).

### Neutrophils egress from the BM to the blood stream after *S*. *aureus* injection near the iLN

**Fig 7:**
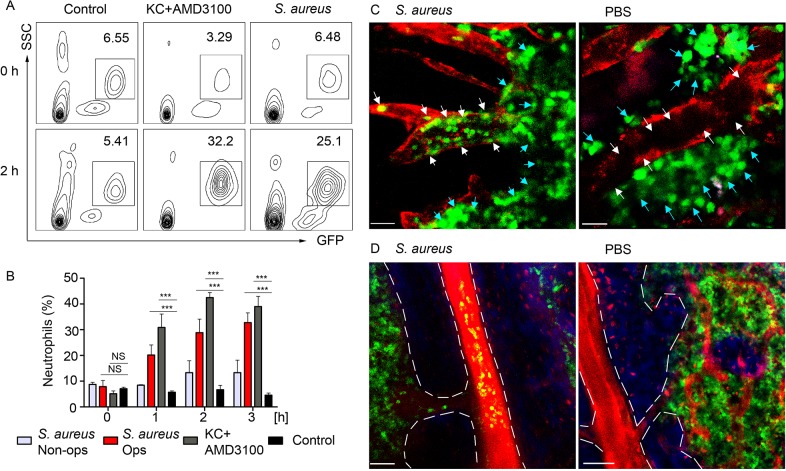
BM neutrophils are mobilized to the blood stream after local immunization. LysM-GFP mice were injected with *S*. *aureus* bioparticles near the iLN. Neutrophil mobilization was analyzed using flow cytometry of the whole blood or TP-LSM of the calvarium bone marrow. Anti VE-cadherin antibody or EB were injected intravenously to outline the blood vessels. (**A**) Flow cytometry plots of GFP^hi^ cells in the blood of mice injected subcutaneously with PBS, intravenously with KC+AMD3100, or subcutaneously with *S*. *aureus* are shown. Results are from 0–2 h after injection and representative of 3 independent experiments. (**B**) Kinetics of neutrophil recruitment in mice injected with opsonized (Ops) or non-opsonized (Non-ops) *S*. *aureus* versus KC+AMD3100 between 0 and 3 h. Data are from flow cytometry analysis of whole blood. N = 3; repeated 3 times. Means ± SEM. (**C**) TP-LSM images of calvarium capillaries (Alexa Fluor 660-conjugated anti-VE-cadherin, red) at 2 h after immunization (left) or PBS injection (right). GFP^hi^ cells (green) within the niche (blue arrows) and within the blood vessels (white arrows) are indicated. Scale bars: 30 μm. (**D**) Images of the central vein in *S*. *aureus* (left) and PBS (right) injected mice 3 h later (S1 Movie). Central vein and vascular niche (EB, red) borders are shown with dashed lines. Scale bars: 50 μm. Images are representative of 5 imaging sessions.

To analyze kinetics of neutrophil recruitment from the BM to the blood in response to a local immunization, we compared the recruitment rates after subcutaneous injection of *S*. *aureus* to those after intravenous KC/AMD3100 injections. Flow cytometry analysis of whole blood revealed a 4-fold increase of the GFP^hi^ cell population in *S*. *aureus* and 10-fold increase in KC+AMD3100 injected mice 2 h after injection (Fig 7A), while neutrophil numbers in the blood of PBS injected control remained at a baseline level. Importantly, neutrophil recruitment to KC+AMD3100 reached plateau 1 h after injection, while peak of neutrophil recruitment after *S*. *aureus* injection was observed between 3 and 4 h after injection (Fig 7B). Furthermore, we found a 3-fold increase in neutrophil recruitment rate in mice injected with opsonized *S*. *aureus* bioparticles comparing to mice injected with non-opsonized bacteria (Fig 7B).

In order to demonstrate mobilization of neutrophils following immunization, we imaged mouse calvarium BM [42] in *S*. *aureus* bioparticle injected LysM-GFP mice. GFP^hi^ cells appeared in the calvarium microvasculature 1 h after immunization. Between 2 and 4 h after immunization GFP^hi^ cells accumulated in the vascular niche, rolling and adhering to the blood vessel wall of the capillaries, while PBS injected mice had only few such cells (Fig 6C and S11 Movie). 3 h post immunization neutrophils started to egress from the BM niche to the blood stream via the central vein (Fig 6D). Only minor neutrophil recruitment to the microvasculature and central vein was observed in PBS injected control mice after 3 h of imaging likely a consequence of the surgical procedure and imaging.

Collectively, these data show that neutrophils are recruited from the BM niche to the vascular niche after subcutaneous injection of *S*. *aureus*. Shortly after, they are released to the circulation in a manner, strongly suggesting cellular uptake of *S*. *aureus* followed by chemoattractant release to the blood stream.

## Discussion

This study provided time-lapse analysis of neutrophil influx to the LN, assessed their role in the development of early humoral response after local *S*. *aureus* immunization and infection, and specified BM origin of LN neutrophils. We have used TP-LSM to show *in vivo* that neutrophils infiltrate the MR and IFZs in bacterial pathogen challenged LN, the areas where B cells migrate and differentiate into PCs. Within the LN, B cells and neutrophils exhibit short- and long-term interactions. The analysis of mice depleted of neutrophils and *in vitro* studies defined a suppressive role of activated neutrophils during the initial LN humoral response likely via their production of TGF-β1.

Neutrophil influx to adjacent LNs during local infection was more abundant and continuous than during immunization with *S*. *aureus* bioparticles or CFA. Since live replicating bacteria were expected to cause stronger innate response, we used 100 fold less CFU than inactivated bacterial particles. In either immunized or infected mice, neutrophils arrived to the LNs with the blood flow, crossed the HEVs and entered the LN parenchyma, migrating along the blood vessels [43] to invade the MR and IFZ. Shown in BLIMP1-YFP mice, neutrophil extravasation and migration occurred mostly within the niches filled with BLIMP1^+^ cells. When co-localized, neutrophils and B cells formed cell-cell interactions and multicellular complexes. The majority of interacting cells were localized along the follicle border, an important site for B cell-T cell interactions [44]. Neutrophils avoided crossing the follicle border and entering the follicle unless laser damage was triggered. Still unidentified molecular signals retain neutrophils outside the CXCL13-rich environment, yet these signals are clearly subordinate to those guiding neutrophils to an inflammatory site. Together with establishing cell-cell interactions, neutrophils actively cleared *S*. *aureus* in the LNs, and swarmed in the IFZ and at the inner surface of the SCS, a process often followed by accelerated neutrophil lysis and NETosis [45].

The effects myeloid cells have on B cell differentiation, maturation and antibody production may be beneficial due to their release of TNF family members BAFF and APRIL [17]; or alternatively, they may be suppressive by their production of prostaglandins [46] or via mechanism not completely understood [47]. We find that LN humoral response increases in neutrophil depleted mice immunized with inactivated *S*. *aureus* or infected with LAC-GFP. Using LAC lysates instead of LAC-GFP verified that IgG and IgM responses were not GFP-specific. As LAC *spa* is an isogenic protein A mutant, it was used to overcome the protein A-dependent non-specific interaction of *S*. *aureus* with the Fc part in antibodies [48]. Therefore, using LAC or LAC *spa* confirmed that in LAC-GFP infected mice detected IgG and IgM responses were *S*. *aureus* specific. Neutrophil influx to immunized LNs could suppress B cell responses in several ways. Most obvious is the removal of antigen [49]. Our data shows presence of LAC in the LNs at day 5 after infection in mice that were depleted of neutrophils during infection. Another possible mechanism may involve SCS macrophages. CD169^+^ macrophages uptake antigens delivered with lymph flow and present them to B cells [50]. Neutrophil swarming and microbial-caused death can lead to the loss of SCS macrophages; and depletion of granulocytes rescues the macrophage layer [4]. Neutrophil influx could damage the SCS macrophages and reduce antigen presentation to B cells resulting in less efficient B cell activation. Supporting this views, our data shows that SCS macrophages more actively participate in bacterial clearance in the LN lacking neutrophils. Finally, the recruitment of activated neutrophils into the LN may directly limit the expansion and/or differentiation of antigen stimulated B cells by producing suppressive cytokines. In this study we show that neutrophils from *S*. *aureus* immunized mice establish cellular protrusions to reach for B cells, and B cells display formation of nanotube-like structures, thus direct interactions may expose B cells to neutrophil-released cytokines. We also show that activated neutrophils secrete TGF-β1, which can suppress antibody production by iLN B cells. In addition, the increased number of B cells in the immunized LN following neutrophil depletion is consistent with either a direct or indirect suppressive effect of neutrophils on humoral response.

A recently published study used BLIMP1-YFP mice to show that myeloid cells shape the formation of the humoral response [47]. Diphtheria toxin-mediated ablation targeted at *Ccr2*-expressing myeloid cells resulted in an enhanced number of antibody secreting cells in the LN. In contrast, depleting Ly6G^+^ cells had little impact on the number of antibody secreting cells in the LN. Our study differs from this study in several ways. First, and most importantly, we depleted neutrophils prior to and during immunization while the other study [47] depleted myeloid cells on day 4 and 6 post immunization. Our data shows that abundant neutrophil influx to the LN occurs as early as 2 to 12 h after immunization or infection. The early arriving neutrophils are likely those that can suppress LN antibody responses. In addition, *S*. *aureus* may recruit more neutrophils into the lymph node and amplify the magnitude of the immune response. Mobilized Ly6G^hi^ cells abundantly co-localized and interacted with BLIMP^+^ cells in the IFZ, the site of the extra follicular antibody response, and later in the MR where PCs are localized. Besides measuring early IgG and IgM production in neutrophil depleted mice, we used the BLIMP1-YFP mice to assess the numbers of emerging antibody secreting cells. Depletion of neutrophils prior to immunization led to increased numbers of GC B cells and a subset of GC B cells expressing BLIMP1. Together, our data suggest direct involvement of neutrophils in control of the humoral response in LNs. Since establishing intercellular contacts with innate cells is a key regulator of antigen-specific B cell differentiation [51], the long-lasting interactions between neutrophils and BLIMP1^+^ cells we observed *in vivo* may represent immune synapse-like formations.

Imaging calvarium BM revealed rapid mobilization of neutrophils in response to *S*. *aureus*. The mobilization of BM neutrophils to an inflammatory site via antagonistically regulated CXCR2/KC and CXCR4/SDF-1α chemokine axes is well documented [52, 53]. However, mature neutrophils can also reside in spleen [17] and migrate between lymphoid compartments during inflammation [9]. Here we show *in vivo* that the egress from the BM precedes neutrophil influx to the iLN. Additionally, our studies indicate that BM derived Ly6G^+^ cells home to immunized iLN when injected intravenously. Our results demonstrate a BM origin for many of neutrophils recruited to the immunized iLN. Whether neutrophils can also be recruited from the spleen remains unclear. The more efficient neutrophil recruitment that occurred with opsonized bioparticles suggests that the cell-mediated uptake of bacteria by macrophages and perhaps other innate cells lining the SCS contributes to the BM neutrophil recruitment.

While neutrophils are imperative as immediate innate defense against *S*. *aureus*, we show that their abundant recruitment to the LNs during local infection or immunization may reduce the efficient development of a specific humoral response. We speculate that the neutrophil influx reduces the exposure of pathogenic antigens to adaptive cells in the LN by phagocytizing bacteria and thus masking the antigen from SCS macrophages. In addition, activated neutrophils exhibit a clear direct suppressive effect on the differentiation of naive LN B cells to antibody secreting cells, which is likely mediated by the release of soluble factors like TGF-β1 and by the direct cell-cell interactions. Thus, while neutrophils may enhance splenic marginal zone B cell responses their recruitment to local LNs is detrimental for local antibody response. As most vaccines are delivered by subcutaneous injection our findings are relevant to immunogen design and the choice of vaccine adjuvants. Adjuvants are necessary to activate innate cells to achieve optimal antibody responses, but as our study indicates there is a potential downside as an overly exuberant neutrophil recruitment will impair antibody responses. Furthermore, directly targeting local TGF-β production would likely augment B cell antibody responses. Our study also raises the possibility that B cell-neutrophil interactions may impact neutrophil function. Future investigations should provide a better understanding of the mechanisms that link the innate and adaptive humoral responses against pathogens such as *S*. *aureus*.

## Materials and Methods

### Mice

All animals were bred and housed under pathogen-free conditions and used according to the guidelines of the Animal Care and Use Committee (NIH). The LysM-GFP mice were provided by Dr. Hyeseon Cho and Dr. Ron Germain (NIAID) with permission from Dr. Thomas Graf (Center for Genomic Regulation, Barcelona, Spain). The BLIMP1-YFP mice were provided by Dr. David Fooksman (Skirball Institute of Biomolecular Medicine, New York, USA) with permission from Dr. Dimitris Skokos (The Rockefeller University, New York, USA). The C57BL/6 GFP-Lifeact mice were provided by Dr. Roland Wedlich-Soldne (Martinsried, Germany). DsRed mice (Jax Stock 006051) were received from Dr. Taha Bat and Dr. Cynthia Dunbar (NHLBI). CD45.1 C57BL/6 mice were purchased from the Jackson Laboratory (Bar Harbor, ME). DsRed and BLIMP1-YFP bone marrow chimeric mice were generated in animal facility within Comparative Medicine Branch (NIH/NIAID) as described in S1 Text. All targeted mouse genes are listed in S1 Table. All experiments were performed using sex and age matched animals, typically between 6 to 10 weeks old.

### *S*. *aureus* variants

*S*. *aureus* LAC clone of strain USA300 (pulsed-field type USA300) was obtained from NARSA (Network on Antimicrobial Resistance in *Staphylococcus aureus*). LAC-GFP clone was generated as a derivative of the USA300 LAC clone constitutively expressing genome-encoded GFP. For the integration of the *gfp* gene, whose DNA sequence was optimized for AT-rich Gram-positive bacteria (*gfpopt*), on the chromosome of LAC clone, first overlap extension PCR was used to create *blaZ-gfpopt*. The constitutively active beta-lactamase promoter was amplified from *S*. *aureus* N315 genomic DNA with primers BlaZFw (ATGCGGATCCCTAACAATAGAAATATAAAACAAAAGC) and BlaZ-GfpRv (AATTCTTCTCCTTTTGACATAATAAACCCTCCGATATTAC) and *gfp-opt* was amplified from plasmid pSW4-GFPopt [54] with BlaZ-GFPFw (GTAATATCGGAGGGTTTATTATGTCAAAAGGAGAAGAATT) and GFPRv (ATGCCTGCAGTTACTTATATAATTCATCCAT). A fusion product of the two PCR fragments was amplified with primers BlaZFw and GFPRv and cloned into plasmid pLL29 [55] BamHI and SalI restriction sites, resulting in plasmid (pLL29-*blaZ-gfpopt*). Plasmid pLL29-*blaZ-gfpopt* was phage-transduced into LAC clone as described previously [55]. The integration of pLL29-*blaZ-gfpopt* into the ϕ11 attachment site of LAC clone was confirmed using primers scv4 (ACCCAGTTTGTAATTCCAGGAG) paired with scv10 (TATACCTCGATGATGTGCATAC) and primer scv8 (GCACATAATTGCTCACAGCCA) paired with scv9 (GCTGATCTAACAATCCAATCCA). Expression of GFPOPT in USA300 LAC clone was confirmed by fluorescence microscopy (excitation/emission at 470 ± 20 nm/ 515 nm, respectively). LAC *S*. *aureus* USA300 LAC *spa* (an isogenic protein A mutant) was a kind gift from Prof. A. Prince (Columbia University, New York, NY).

### Bacteria culture and CFU counts

Glycerol stocks of *S*. *aureus* USA300-derivative LAC-GFP were grown to mid-exponential growth phase (for min of 2 h) in 50 ml of TSB at 37 °C with shaking at 180 rpm. Bacteria were harvested and washed and resuspended in sterile PBS prior to injections. CFU counts from infected LNs were performed as described [56]. Shortly, mice were euthanized and LNs were harvested. One LN of each mouse was placed into a 2-ml tube containing 1 ml of sterile PBS with 500 mg of 2 mm borosilicate glass beads (Sigma). The LNs were homogenized in a Fast Prep bead beater (Thermo Savant) at 6 m/s for 20 s. The homogenates were diluted in PBS, plated onto TSB plates, and incubated overnight at 37°C for CFU counting.

### Antigens and immunization

CFA containing heat killed *M*. *tuberculosis* (H37Ra), 0.85 mL paraffin oil and 0.15 mL mannide monooleate (Sigma Aldrich) was injected in amount of 50 μl per iLN. *S*. *aureus* Bioparticles were purchased from Life Sciences (Molecular Probes, Cat #. S2851, S23371, S23372). The reagent consists of heat-killed bacteria Wood 46 strain without protein A, unlabeled or fluorescently labeled with either Alexa Fluor 488 or Alexa Fluor 594. Alexa Fluor dyes of *S*. *aureus* Bioparticle conjugates are bound to the surface of bacterial cells, but not internalized. Prior to use, the Bioparticles were coated with *S*. *aureus* opsonizing reagent that contains rabbit polyclonal IgG antibodies specific for *S*. *aureus* and RIA-grade bovine serum albumin to block nonspecific binding (Molecular Probes, Life Sciences, Cat #. S2860). Opsonized *S*. *aureus* bioparticles were injected in amount of 2 x 10^7^ bacterial particles per mouse (1 x 10^7^ per iLN). NP-KLH (Biosearch Technologies) precipitated in Alum (Thermo Scientific) was given at amount of 25 μg per iLN. Sheep red blood cells (SRBC, Lonza) were administered in amount of 1 x 10^8^ cells per iLN. *S*. *aureus* USA300-derivative LAC-GFP was injected at 1–3 x 10^5^ CFU per mouse (0.5–1.5 x 10^5^ CFU per iLN) in 200 μL of sterile PBS. All antigens were injected subcutaneously within 1 cm of the adjacent iLN (S1A Fig).

### Kinetics of neutrophil recruitment

Neutrophil influx to the iLN was monitored using TP-LSM or confocal microscopy between 0 and 24 h; and in the iLN, blood, draining LNs and spleen using flow cytometry between 0 to 24 h, or days 1 to 5 after immunization. Whole blood was collected from mouse tails (tail snip), and iLN cells or splenocytes were isolated from euthanized mice. LysM-GFP cells were analyzed directly, and C57BL/6 cells were immunostained using fluorochrome conjugated anti-Ly6G/ (clone RB6-8C5),-B220,-CD11c,-GL7,-Fas,-CD4,-CD8,-CD169, and—F4/80 antibody (eBioscience). Flow cytometry was performed using FACS Canto II flow cytometer with FACS Diva 6.2 software (BD Biosciences).

### Cell isolation and adoptive transfer

Immune cell subsets were isolated using magnetic negative selection system with dynabeads M-280 streptavidin and magnetic particle concentrator (Invitrogen) as previously described [57]. BM derived neutrophils were isolated from mouse femur and tibia using anti B220,-CD38,-CD138,-CD11c,-CD4 and-CD8; and B cells were isolated from mouse spleen by using anti CD11c,-Gr-1,-CD4 and-CD8 biotinylated antibody (BD Pharmingen). Isolated cells were cultured in complete lymphocyte medium (DMEM supplemented with 10% FBS, 25 mM HEPES, 50 μM β-ME, 1% Pen/Strep/L-Glu and 1% Sodium Pyruvate) in humidified CO_2_ incubator. Cells were allowed to recover for 30 min, and labeled with CellTracker™ Blue, CMAC, CellTracker™ Green, CFDMA or CellTracker™ Red, CMTPX (Molecular Probes, Invitrogen) according to the manufacturer’s protocol. Labeled cells were administered intravenously into recipient mice (5 x 10^6^ cells per mouse) via tail vein injection, and imaged between 16 and 24 h later.

### Confocal microscopy

Isolated mouse LNs were sliced into 250 μm sections using Leica VT1000 S Vibrating Blade Microtome (Leica Microsystems). Live cell imaging of immunostained sections was performed using Leica SP8 inverted 5 channel confocal microscope equipped with a motorized stage and 2 HyD ultra-sensitive detectors (Leica Microsystems). Images of whole LNs were tiled using Leica Application Suite (Leica Microsystems) and processed using Imaris (Bitplane) software. For live cell imaging, BM-isolated neutrophils and LN-isolated B cells were cultured for 2 h on ICAM-1 + VCAM-1 + KC (Recombinant Mouse ICAM-1/CD54 Fc Chimera, CF; Recombinant Mouse VCAM-1/CD106 Fc Chimera, Recombinant Mouse CXCL1/KC CF; R&D Systems) coated glass-bottom dishes (No 1.5 coverglass; MatTek). Live cells were stained in complete medium with fluorescently labeled anti-Ly6G and anti-B220 correspondingly (BD Pharmingen). Confocal imaging was performed using Leica SP8 equipped with incubation chamber (CO_2_, 37°C) for live cell imaging (Pecon). Images were processed using Imaris (Bitplane) software. Detailed description of confocal microscopy setup is provided in supporting information (S2 Text).

### Epifluorescent microscopy

Immunized mice were injected intravenously with 1% EB solution in PBS (Evans blue dye, Sigma Aldrich) at 1 ml/kg. Mice were euthanized, iLNs exposed on a skin flip, kept moisturized with PBS and imaged immediately after exposure. Fluorescent and bright field images of intact mouse iLNs were collected using motorized stereomicroscope Leica M205 (Leica Microsystems) equipped with 1x objective. GFP/YFP were excited at 488 nm and EB at 561 nm. Images were processed using Leica Application Suite (Leica Microsystems) and Imaris (Bitplane) software.

### Intravital two-photon laser scanning microscopy (TP-LSM)

All imaging experiments were performed at Biological Imaging Section (NIH, NIAID) using Leica SP5 inverted confocal microscope (Leica Microsystems) equipped with dual Mai Tai lasers as previously described [57]. Mouse surgery for imaging the iLN was performed according to the Cold Spring Harbor protocol [58] modified for the inverted microscope setup. For imaging neutrophil recruitments from the BM mice were injected intravenously with KC+AMD3100 (AMD 3100 octahydrochloride; Recombinant Mouse CXCL1/KC CF; R&D Systems). Mouse calvarium BM was imaged as described [42], using upright microscope setup and a custom-made stage with the head holder (NIH Division of Scientific Equipment and Instrumentation Services). Post-acquisition image processing was performed using ImageJ (National Institutes of Health), Imaris (Bitplane) and Huygens (Scientific Volume Imaging) software. Detailed description of the imaging technique is provided in supporting information (S3 Text).

### Neutrophil depletion and ELISA with LN derived B cells

Neutrophils were depleted *in vivo* as described [59] using anti Ly6G functional grade antibody 1A8 (eBioscience/BioLegend). Briefly, animals were injected intraperitoneally with 100 μg of isotype control rat immunoglobulin G (eBioscience/BioLegend) or 1A8 antibody at days -1, 0 and 1 of immunization. Efficiency of depletion was monitored by flow cytometry analysis or by TP-LSM, and typically represented > 90% in blood, spleen and draining LNs. At days 5 to 7 of immunization (6 to 8 of depletion) mice were sacrificed and the iLNs harvested. B cells isolated from a single iLN were cultured in complete lymphocyte medium for 72 h, and the supernatants were collected. Antibody concentration in the supernatants was measured with commercial ELISA kits (Mouse IgG total ELISA Ready-SET-Go, Mouse IgM total ELISA Ready-SET-Go; eBioscience) according to the manufacturer's protocol. LAC-specific IgG and IgM were measured using plates coated with bacterial lysates. Total lysates from LAC and LAC *spa* were prepared as previously described [60] with the following modifications. Bacteria were grown as above, pellets were resuspended in 1 ml of sterile PBS and incubated 30 minutes at 37 °C in the presence of Halt protease inhibitor single use cocktail (Thermo Scientific) and lysostaphin. The digested lysates were transferred to 2-ml Lysing Matrix B vials (MPbio) and homogenized in a Fast Prep bead beater (Thermo Savant) at 6 m/s for 20 s. Protein concentrations were determined with the Quant-iT assay kit (Life Technologies). IgG and IgM specific for NP-KLH were measured by ELISA using plates coated with NP-KLH. For antigen-specific ELISAs plates were coated at protein concentrations 10 μg/mL (LAC or LAC *spa* lysates) and 1 μg/mL (NP-KLH) in PBS overnight and blocked with 1% BSA in PBS.

### Neutrophil and B cell activation *in vitro*

Neutrophils were isolated from the BM and cultured in for 24 h in presence of 2 μg/mL LPS (from E. coli, Serotype R515 (Re), TLR grade, ENZO Life Sciences) or 1 x 10^6^ particles/mL *S*. *aureus* bioparticles. Supernatants were added to freshly isolated LN B cells. Isolated iLN B cells were cultured in complete lymphocyte medium at initial concentration between 5 x 10^5^ to 2 x 10^6^ cells/mL. B cells and neutrophils were added to the cultures in ratio 10 B cells: 1 neutrophil, for 5 days in presence of LPS or *S*. *aureus* bioparticles. Alternatively, supernatants from either activated or non-activated neutrophil cultures (25% of culture medium) were added to B cells. Final concentration of LPS was 2 μg/mL, and *S*. *aureus* 1 x 10^6^ particles/mL in all cultures. Neutralizing anti TGF-β1 (TGF-beta 1/1.2 Polyclonal antibody; R&D Systems) were added at final concentration 1 μg/mL, and TGF-β1 (50 ng/mL). TGF-β1 in neutrophil and B cell cultures was measure using commercial ELISA kit (eBioscience). IgM and IgA levels in the supernatants were measured with commercial ELISA kits (eBioscience) according to the manufacturer's protocol.

### Ethics statement

The animal experiments and protocols were performed according to the regulations of NIAID Division of Intramural Research Animal Care and Use Committee (DIR ACUC). Animal Study Proposal LIR 16 entitled “Analysis of Innate Immune Function in Mice” that covers this work was approved by the NIAID DIR ACUC on Dec 1^st^, 2011 as the initiation date, and has been reviewed annually. The NIAID DIR ACUC as a part of the NIAID DIR Animal Care and Use Program, as part of the NIH Intramural Research Program (IRP), complies with all applicable provisions of the Animal Welfare Act and other Federal statutes and regulations relating to animals; and is guided by the "U.S. Government Principles for the Utilization and Care of Vertebrate Animals Used in Testing, Research, and Training". The policies, procedures and guidelines for the NIAID IRP are explicitly detailed in NIH Policy Manual 3040–2, “Animal Care and Use in the Intramural Program” (PM 3040–2) and the NIH Animal Research Advisory Committee Guidelines (ARAC Guidelines) that are posted on the NIH Office of Animal Care and Use public website at: http://oacu.od.nih.gov.

### Statistical analysis

The statistical significance was evaluated by subjecting the data to a Student's t-test using GraphPad software. Values are presented as means ± SD or means ± SEM as indicated. *, P<0.05; **, P<0.01; ***, P<0.001.

## Supporting Information

S1 Fig Neutrophil recruitment to CFA immunized iLN. (**A**) Schematic of mouse injection site. (**B—E**) C57BL/6 mice were immunized with CFA. Kinetics of leukocyte recruitment to the blood stream and to the iLNs was analyzed by flow cytometry. Representative plots of the Ly6G^hi^/CD11b^hi^ population in mouse (B) whole blood and (C) iLN at 0, 2, 4, and 12 h after CFA injection are shown. (D) Neutrophil numbers in the iLN 0, 2, 4, and 24 h after CFA immunization. N = 2 mice/4 iLNs. Data was reproduced 3 times. Means ± SD. (E) Lymphocyte populations present in the iLN of mice at 24 h after immunization. Percent and absolute cell numbers are shown. 3 mice/6 iLNs per group were analyzed. Results represent 3 independent experiments. (**F**) TP-LSM images of B cell follicle (red) in naïve LysM-GFP iLN with induced laser damage (pink, blue arrowhead) at 0 and 30 min are shown. Blood vessels are visualized with EB (gray). Collagen fibers, blue. Scale bars, 50 μm. (**G**) Time-lapse series of images showing steps of neutrophil (GFP^hi^, green) swarming to the laser damaged site in B cell follicle over the course of 60 min. Scale bars, 35 μm. Related to Fig 1.
(TIF)

S2 Fig Neutrophil recruitment to *S*. *aureus* immunized iLN and interactions with B cells. (**A**, **B**) Kinetics of neutrophil recruitment to the draining LNs and spleen after local immunization of the iLN with *S*. *aureus* was measured at 0, 4 and 12 h after immunization using flow cytometry. Analysis of Ly6G^+^/CD11b^+^ population in the (A) axillary, superficial cervical or inguinal LNs and (B) spleen after *S*. *aureus* injection is represented. 3 mice/6 LNs per group were analyzed Means ± SD (**C**, **D**) Time-lapse series of TP-LSM images showing formation of (C) short-term or (D) long-lasting interactions between neutrophils (green) and B cells (red) in immunized iLN *in vivo*. Scale bars: 5 μm. (**E, F**) Flow cytometry analysis of the cell populations that acquired *S*. *aureus* in the iLN 12 h after immunization. (E) Upper gate shows S. aureus^+^/Ly6G^+^ population while the lower gate shows *S*. *aureus* ^*+*^ */*Ly6G^-^ population. (F) The percentages of Ly6G^+^, CD169^+^, F4/80^+^ or CD11c^+^cells that engulfed *S*. *aureus* (chart). Data is representative of 3 independent experiments. 2 mice mice/4 iLNs per group analyzed. Related to Fig 2.
(TIF)

S3 Fig Neutrophils infiltrate iLN after local LAC-GFP infection. DsRed chimeric mice were injected with PBS or infected with LAC-GFP locally, near the iLN, and analyzed using flow cytometry or TP-LSM 24 h after injections. (**A**) Flow cytometry analysis of whole blood. Granulocyte gates in LAC-GFP infected mice (upper left) or PBS-injected control (lower left) are indicated with circles. DsRed^hi^ population (upper right) from live cell gate and Ly6G^+^/CD11b^+^ population (lower right) from dsRed^hi^ gate (marked with red) in LAC-GFP infected mice are shown. (**B**) Flow cytometry analysis of iLN cells. DsRed [hi] and [med/lo] gates in LAC-GFP infected mice (upper left) are shown. Ly6G^+^/CD11b^+^ population (upper right) from DsRed^hi^ gate (marked with red) and B220^+^ population (lower left) from dsRed^med/lo^ gate (marked with purple) in LAC-GFP infected mice are shown. DsRed [hi] and [med/lo] gates in PBS-injected control (lower left) are shown. (A, B) Representative plots of 3 mice per group analyzed. (**C**) ILNs in PBS control mice (left panel) and in 24 h-infected mice (right panel) are shown. Neutrophils (dsRed^hi^, red). LN borders and follicular borders are shown with white dashed lines. B cell follicles (B); interfollicular zones (IFZ) are labeled. Scale bars: 50 μm. (**D**) LysM-GFP mice were injected with PBS or immunized near the inguinal LN with SRBC. Labeled B cells were adoptively transferred 24 h prior to imaging. ILNs in PBS control mice (left panel) and in immunized mice at day 3 after immunization (right panel) are shown. Neutrophils (GFP^hi^, green); B cells (CMTMR, red); blood vessels (Evans Blue, gray). LN borders are shown with white dashed lines; B cell follicles (F) and HEVs (HEV) are labeled. Scale bars: 50 μm; Z = 50 μm. (A-B) Representative images of 3 experiments. Related to Fig 3.
(TIF)

S4 Fig F-actin accumulates faster during neutrophil interactions with B cells than during phagocytosis of *S*. *aureus* particles. Lifeact-GFP neutrophils and B cells were co-cultured on ICAM-1+VCAM-1+KC coated surface and imaged using confocal microscopy. B cells were immunostained with anti-MHCII antibody. *S*. *aureus* bioparticles were added to the co-cultures and cells were imaged immediately (A-C) or after 2 h (D-E). (**A**) A confocal image of neutrophils (green) phagocytizing *S*. *aureus* (red). 3 regions of interest (cells) for quantitative analysis are indicated with squares. Scale bar: 15 μm. (**B**) Time-lapse series of confocal images showing steps of *S*. *aureus* uptake by a single neutrophil. F-actin clustering during the uptake is shown with red arrows. Scale bar: 7 μm; time is relative. (**C**) Profiles of GFP mean fluorescence in 3 cells indicated as regions of interest in (A). Changes in a curve slope shown with arrows. (**D**) A confocal image of neutrophils (green) interacting with B cells (blue). 3 regions of interest are indicated with squares. Scale bar: 10 μm. (**E**) Time-lapse series of confocal images showing steps of cell-cell contact formation between a neutrophil and a B cell. F-actin clustering during the ionteraction is shown with white arrows. Scale bar: 5 μm; time is relative. (**F**) Profiles of GFP mean fluorescence in 3 cells indicated as regions of interest in (D). Changes in curve slopes shown with arrows. Related to Fig 4.
(TIF)

S5 Fig LN B cells increase antibody production in neutrophil-depleted mice. (**A**) Flow cytometry plots of the whole blood from PBS control or *S aureus* immunized mice, injected with isotype control antibody or depleting antibody clone 1A8 24 h after depletion. (**B**) Flow cytometry plot of iLN Ly6G^hi^ cell population from PBS control or *S aureus* immunized mice, injected with isotype control antibody or 1A8 antibody; 24 h after depletion. Data is representative of experiments using 2 mice/4 iLNs mice per group; repeated 3 times. (**C**) TP-LSM of iLN in neutrophil-depleted mice. Scale bars, 35 μm. N = 3. (**D**) Flow cytometry analysis of B and T cell populations in the iLN of neutrophil-depleted or isotype control mice 3 days after immunization. The data are shown as cell numbers. N = 4 iLNs; 3 repeats. Means ± SEM. (**E**) ELISA of IgG and IgM produced by B cells isolated from a single iLN from mice injected with isotype control or 1A8 antibody and immunized with *S*. *aureus* bioparticles (**F**) ELISA of total IgG and IgM produced by 1 x 10^5^ iLN B cells isolated from mice injected with isotype control or 1A8 antibody and immunized with *S*. *aureus* bioparticles. (E, F) B cells harvested at day 6 after immunization, cultured for 3 days. N = 4 iLNs; 3 repeats. Means ± SD. (**G**) CFU counts in 24 h cultures of LNs isolated from LAC-infected mice that were injected with PBS, isotype control antibody or 1A8 antibody. Number of CFU grown from one LN is shown to the left. Percent of lymph nodes that cleared LAC infection completely (0 CFU) versus those that contained LAC (CFU growth) is shown to the right. Single experiment. N = 5. (**H**) ELISA of total IgG and IgM produced by iLN B cells from mice injected with isotype control or 1A8 antibody and immunized with SRBC (**I**) ELISA of NP-KLH specific IgG and IgM produced by iLN B cells from mice injected with isotype control or 1A8 and immunized with NP-KLH. (G, H) B cells were harvested at day 7 after immunization and cultured for 3 days. N = 4 iLNs; 3 repeats. Means ± SEM. (**J**) B cells were isolated from LNs, and activated in vitro with *S*. *aureus* bioparticles. ELISA of total IgM and IgA produced by total iLN B cells cultured with (B + N) or without neutrophils (B cells) for 5 days. Non-activated (NA) B cell/Neutrophil co-cultures were used as a base-line control. (**K**) B cell numbers in *S*. *aureus*-activated cultures as shown by flow cytometry analysis at days 0 and 5. (**L**) IgM and IgA fold decreases calculated for total LN B cell cultures (left) and for 1 x 10^6^ B cells (right) in presence of neutrophils. (J—L) N = 4 iLNs; 3 repeats. Means ± SEM. Related to Fig 5.
(TIF)

S6 Fig Localization of neutrophils in *S aureus* bioparticle immunized iLN in BLIMP1-YFP mice at day 7 after immunization. (**A**) Flow cytometry analysis of B220^+^ cell population in the iLN of neutrophil-depleted or isotype control mice 7 days after *S*. *aureus* bioparticle immunization. B220^+^ total cell number and percentage within the lymphocyte gate are shown. N = 4 iLNs; 3 repeats. Means ± SEM. (**B-D**) Flow cytometry analysis of Ly6G^+^/CD11b^+^ cell population in the iLN 7 days after immunization. (B) Representative plots of Ly6G^+^/CD11b^+^ population in PBS-injected or *S*. *aureus* immunized mice. (C) Ly6G^+^/CD11b^+^ percentage and total cell numbers within live cell gate are shown. (D) Percentages and total cell numbers of endogenous (WT) and adoptively transferred neutrophils (DsRed) are shown. Adoptive transfer was performed 12 h prior to analysis. N = 4 iLNs; 3 repeats. Means ± SEM. (**E**) Tiled confocal image of *S*. *aureus* immunized BLIMP1-YFP iLN at day 7 after immunization. TZ (T), IFZ (IF), medulla (MR), and LN follicles are labeled. Perivascular niches filled with BLIMP1-YFP^+^ cells are indicated with white arrows. Enlarged image of the IFZ (white square) is shown to the right. Immunostaining: B220 (blue), LYVE-1 (purple), Ly6G (gray). Endogenous: BLIMP1-YFP (yellow-green). Related to Fig 6.
(TIF)

S1 Movie Neutrophils infiltrate CFA immunized LN. TP-LSM acquired images of the inguinal LN from a LysM-GFP mouse immunized with CFA 2 h previously and, which had received B cells (red) by adoptive transfer. Neutrophils (green) infiltrate LN stroma (left panel) arriving from the blood vessels (right panel) visualized via i.v. injection of Evans Blue (gray). Scale bars: 100 μm (left), 50 μm (right); Z = 50 μm. Time-lapse movie was generated using Imaris at 20 frames per second. Related to Fig 1.
(MOV)

S2 Movie Neutrophils swarm in laser damaged site inside a B cell follicle. Laser damage was induced inside a B cell follicle of the iLN of a LysM-GFP mouse by applying 50% of the MP laser power at zoom 25 for 3 sec. The LN was imaged over the next 60 minutes. B cells (red) were adoptively transferred the day before to outline the B cell follicles. Evans Blue was injected intravenously prior to imaging to visualize blood vessels (gray). Neutrophils (GFP^hi^, green) migrating within the interfollicular zone and along the follicular border are recruited into the follicle to the site of laser damage (pink). Scale bar: 50 μm; Z = 50 μm. Z stacks were acquired every 12 sec and time-lapse movie was generated at 20 frames per second using Imaris. Related to Fig 1.
(MOV)

S3 Movie Neutrophils infiltrate *S*. *aureus* immunized LN and swarm. LysM-GFP mice were immunized near the inguinal LN with fluorescent *S aureus* bioparticles (red). The iLNs were imaged using TP-LSM starting at 2 h after immunization. Mobilization: neutrophils (GFP^hi^, green) infiltrate the LN arriving from the blood vessels (blue arrows) and migrating toward the SCS where bacteria arrived with the lymph flow (red arrows). Events between 2 and 3 h after immunization are shown. Scale bar: 100 μm; Z = 50 μm. Swarming: neutrophils (GFP^hi^, green) loaded with bacterial particles travel through the LN stroma and swarm. Events between 3 and 4 h after immunization are shown. Scale bar: 50 μm; Z = 50 μm. Z stacks for time-lapse videos were acquired every 12 sec for 30 min each and movies were generated at 20 frames per second using Imaris. Related to Fig 2.
(MOV)

S4 Movie B cells localize in the follicle and Neutrophils at the follicle border in immunized iLN. TP-LSM time-lapse video of the iLN in a LysM-GFP mouse immunized with unlabeled *S*. *aureus* bioparticles 12 h before imaging. Adoptively transferred dsRed B cells (red) migrate within the follicle. LysM-GFP neutrophils (green), recruited to immunized iLN, localize in IFZ and around B cell follicle. Blood vessels are outlined by intravenous injection of Evans Blue (gray). Scale bar: 100 μm; Z = 50 μm. Z stacks for time-lapse video were acquired every 12 sec for 60 minutes and the movie was generated at 20 frames per second using Imaris. Related to Fig 2.
(MOV)

S5 Movie Interactions between neutrophils and B cells in immunized inguinal LN. A LysM-GFP mouse was injected subcutaneously with unlabeled *S*. *aureus* bioparticles and the adjacent iLN was imaged using TP-LSM 12 h after immunization. Mobilized to the iLN neutrophils (GFP^hi^, green) form intercellular interactions with B cells (DsRed) around a blood vessel (Evans Blue, gray), near the follicle border. White arrows mark transient (less than 30 sec) and white circles long-lasting (over 30 min) neutrophil-B cell interactions. Scale bar: 50 μm; Z = 60 μm. Recorded over 60 min. Z stacks were acquired every 12 sec and time-lapse movie was generated at 20 frames per second using Imaris. Related to Fig 2.
(MOV)

S6 Movie Short-lived interactions between neutrophils and CD4^+^ T cells at the T-B border. A LysM-GFP mouse was injected subcutaneously with *S*. *aureus* bioparticles and the adjacent iLN imaged using TP-LSM 12 h after immunization. DsRed CD4^+^ T cells were adoptively transferred 24 h prior to imaging. Mobilized to the follicle border neutrophils (GFP^hi^, green) form transient cell-cell contacts (white arrows) with CD4^+^ T cells (red) migrating from T cell zone toward the T-B border. DCs (GFP^med^, green) form long-term interactions with CD4^+^ T cells (white circles). Scale bar: 30 μm; Z = 60 μm. Z stacks were acquired every 12 sec and time-lapse movie was generated at 20 frames per second using Imaris. Related to Fig 3.
(MOV)

S7 Movie Neutrophils infiltrate adjacent iLN after a local LAC-GFP infection. DsRed chimeric mice were imaged using TP-LSM after subcutaneous injections of fluorescent *S*. *aureus* clone LAC-GFP (green). ILNs at 2 h (left panel) and 12 h (right panel) after infection are shown. Neutrophils (dsRed^hi^, red) arrive to the LN and infiltrate first interfollicular areas (left) then SCS and TZ (right). Scale bars: 50 μm; Z = 50 μm. Z stacks of were acquired every 12 sec for 60 minutes and a time-lapse movie was generated at 20 frames per second using Imaris. Related to Fig 3.
(MOV)

S8 Movie Neutrophils loaded with LAC-GFP migrate in the stroma of an infected iLN. The iLN in dsRed chimeric mice was imaged using TP-LSM after subcutaneous injection of LAC-GFP (green). Neutrophils are distinguished as dsRed^hi^ (bright red) highly migratory cells. Neutrophils that travel across the stroma of infected iLN often carry *S*. *aureus* (A, B), while some of recruited cells are still free of bacteria (C, D). Scale bars: 10 μm; Z = 30 μm. Z stacks were acquired every 12 sec for 60 minutes and time-lapse movies were generated at 20 frames per second. Migrating neutrophils were tracked post-acquisition (white circles) and their tracks outlined using Imaris. Related to Fig 3.
(MOV)

S9 Movie F-actin clustering during the intercellular interactions. Lifeact-GFP bone marrow derived neutrophils (green) were co-cultured with B cells (blue), and opsonized *S*. *aureus* bioparticles (red) were added to the culture. Phagocytosis of *S*. *aureus* by neutrophils and formation of intercellular contacts between activated neutrophils and B cells was recorded using live-cell confocal imaging. F-actin clustering in neutrophils during intercellular interactions is indicated with white arrows. Scale bars: 10 μm; Z = 20 μm. Single images were taken every 9 seconds and processed as a time-lapse movie at 20 frames per second using Imaris. Related to Fig 4.
(MOV)

S10 Movie Adoptively transferred dsRed neutrophils migrate within perivascular niches formed by BLIMP1^+^ cells in *S*. *aureus* immunized iLN. BLIMP1-YFP mice were immunized with *S*. *aureus* bioparticles. The iLNs were imaged using TP-LSM at day 7 after immunization and 24 h after the adoptive transfer of BM derived dsRed neutrophils. Neutrophils (red) migrate within the inner perimeter of perivascular niches formed by BLIMP1^+^ cells (green). Along their tracks (white lines), neutrophils interact with BLIMP1^+^ cells. Other mobilized neutrophils (red) are arrested within the niche. Scale bar: 50 μm; Z = 35 μm. Z stacks were acquired every 12 sec, time-lapse movie was generated at 20 frames per second, and cell tracks of migrating neutrophils were outlined using Imaris. Related to Fig 6.
(MOV)

S11 Movie Recruitment of neutrophils from the bone marrow to the blood stream after local immunization with *S*. *aureus*. LysM-GFP mice were injected intradermally with *S*. *aureus* bioparticles and the calvarium bone marrow was imaged using TP-LSM between 1 and 3 h after immunization. Neutrophils (GFP^hi^, green) are mobilized to the capillaries 2 h after *S*. *aureus* immunization (right panel) in contrast to PBS injected control (left panel). Evans Blue injected intravenously outlines blood vessels (red). Vascular niche capillaries were distinguished by their morphology. Scale bars: 100 μm; Z = 50 μm. Z stacks were acquired every 9 sec, time-lapse movie was generated at 20 frames per second using Imaris, and processed using Huygens (SVI) software. Related to Fig 7.
(MOV)

S1 Table ID Numbers in Mouse Genome Informatics database (MGI). List of targeted mouse genes with gene symbols, synonyms, full names and MGI ID numbers are provided in alphabetical order.
(DOCX)

S1 Text Supporting material and methods. Generation of bone marrow chimeric mice is described.
(DOCX)

S2 Text Supporting material and methods. Details of confocal microscopy setup and live LN sectioning are provided.
(DOCX)

S3 Text Supporting material and methods. Intravital two**-**photon laser scanning microscopy (TP-LSM) setup is described in detail.
(DOCX)
