# Supplementary figures and images for: Neutrophil Recruitment to Lymph Nodes Limits Local Humoral Response to Staphylococcus aureus
Source: PLoS Pathog. 2015 Apr 17;11(4):e1004827. doi: 10.1371/journal.ppat.1004827 (PMC4401519; doi:10.1371/journal.ppat.1004827)

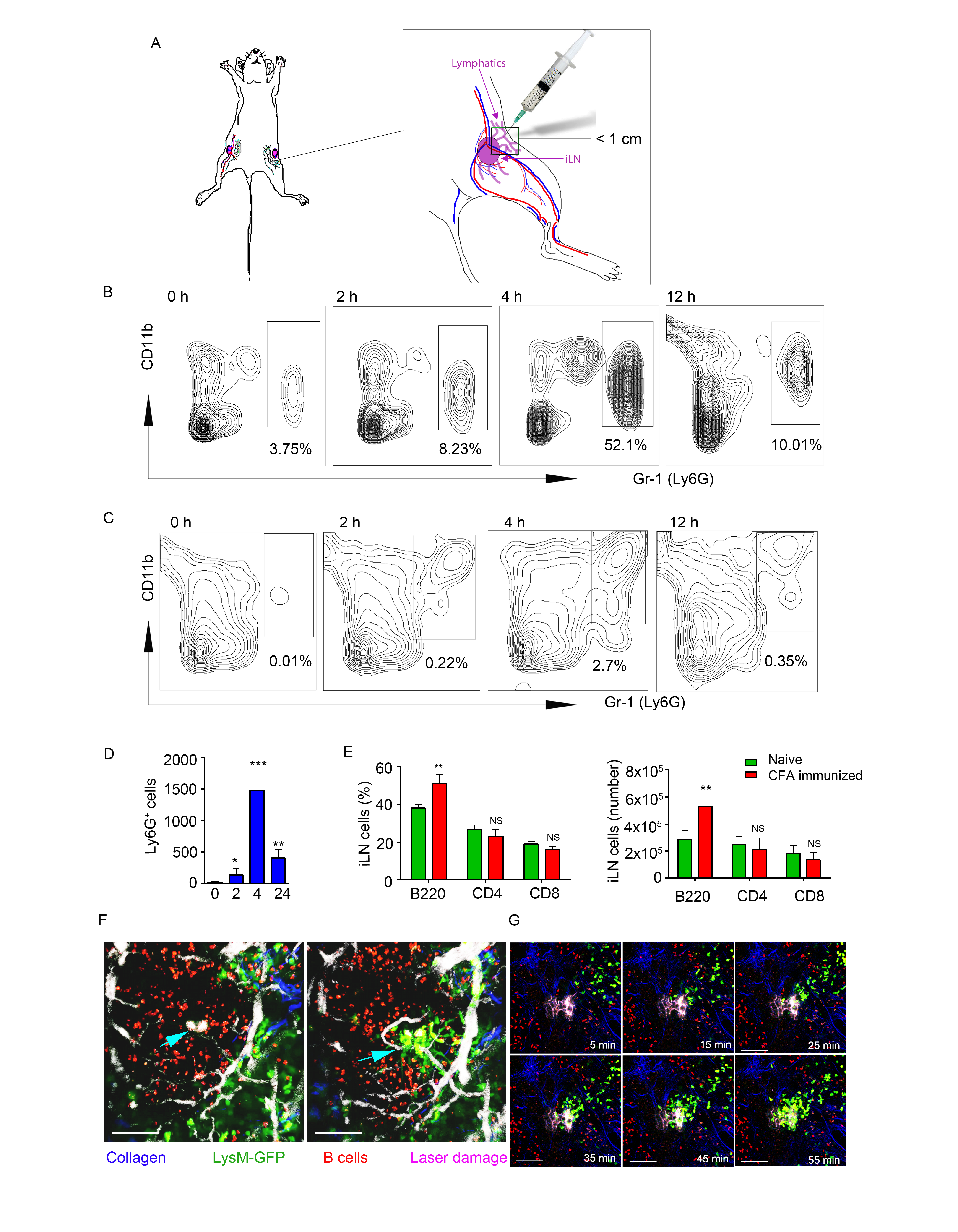

Supplement: S1 Fig — (A) Schematic of mouse injection site. (B—E) C57BL/6 mice were immunized with CFA. Kinetics of leukocyte recruitment to the blood stream and to the iLNs was analyzed by flow cytometry. Representative plots of the Ly6Ghi/CD11bhi population in mouse (B) whole blood and (C) iLN at 0, 2, 4, and 12 h after CFA injection are shown. (D) Neutrophil numbers in the iLN 0, 2, 4, and 24 h after CFA immunization. N = 2 mice/4 iLNs. Data was reproduced 3 times. Means ± SD. (E) Lymphocyte populations present in the iLN of mice at 24 h after immunization. Percent and absolute cell numbers are shown. 3 mice/6 iLNs per group were analyzed. Results represent 3 independent experiments. (F) TP-LSM images of B cell follicle (red) in naïve LysM-GFP iLN with induced laser damage (pink, blue arrowhead) at 0 and 30 min are shown. Blood vessels are visualized with EB (gray). Collagen fibers, blue. Scale bars, 50 μm. (G) Time-lapse series of images showing steps of neutrophil (GFPhi, green) swarming to the laser damaged site in B cell follicle over the course of 60 min. Scale bars, 35 μm. Related to Fig 1. (TIF) [file ppat.1004827.s001.tif]

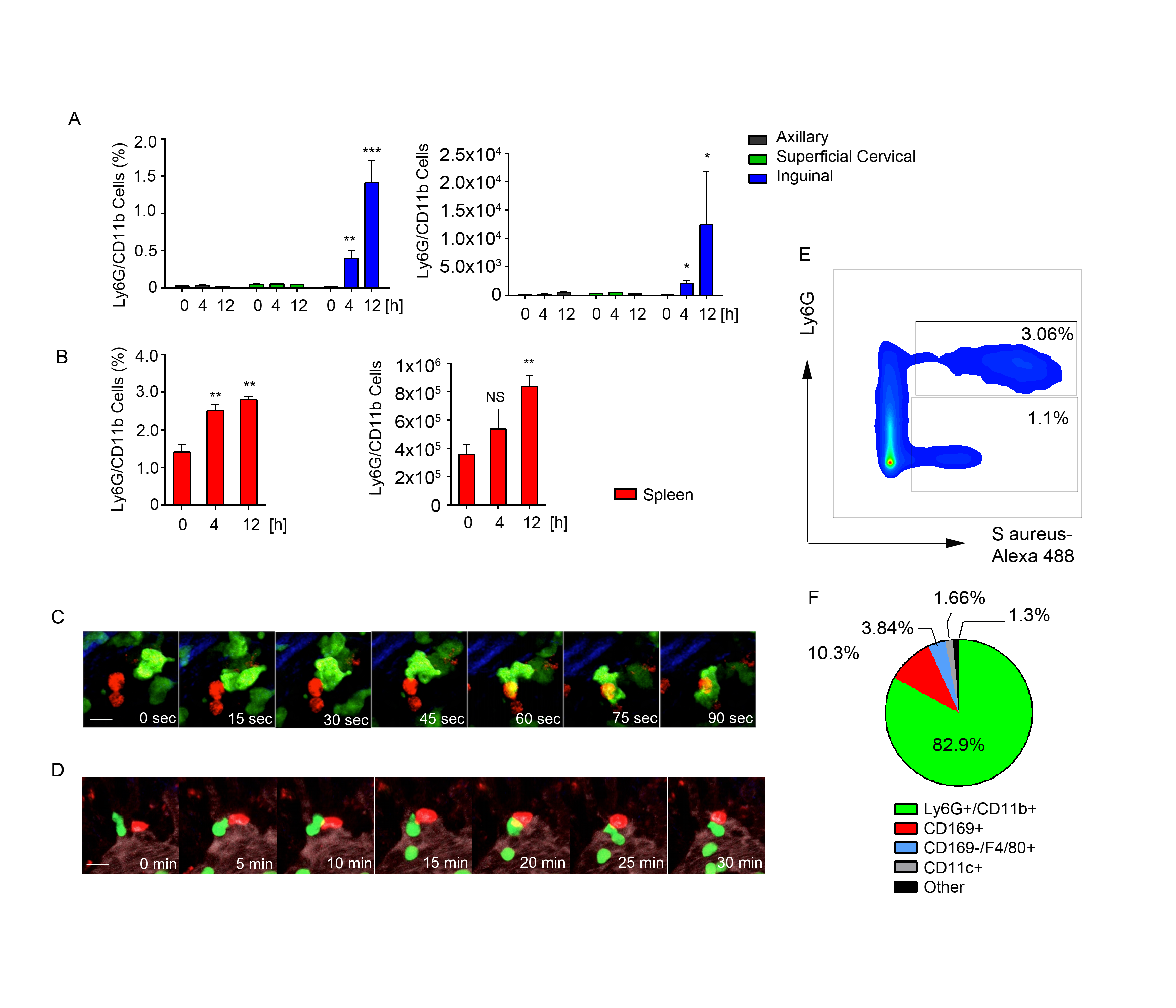

Supplement: S2 Fig — (A, B) Kinetics of neutrophil recruitment to the draining LNs and spleen after local immunization of the iLN with S. aureus was measured at 0, 4 and 12 h after immunization using flow cytometry. Analysis of Ly6G+/CD11b+ population in the (A) axillary, superficial cervical or inguinal LNs and (B) spleen after S. aureus injection is represented. 3 mice/6 LNs per group were analyzed Means ± SD (C, D) Time-lapse series of TP-LSM images showing formation of (C) short-term or (D) long-lasting interactions between neutrophils (green) and B cells (red) in immunized iLN in vivo. Scale bars: 5 μm. (E, F) Flow cytometry analysis of the cell populations that acquired S. aureus in the iLN 12 h after immunization. (E) Upper gate shows S. aureus+/Ly6G+ population while the lower gate shows S. aureus + /Ly6G- population. (F) The percentages of Ly6G+, CD169+, F4/80+ or CD11c+cells that engulfed S. aureus (chart). Data is representative of 3 independent experiments. 2 mice mice/4 iLNs per group analyzed. Related to Fig 2. (TIF) [file ppat.1004827.s002.tif]

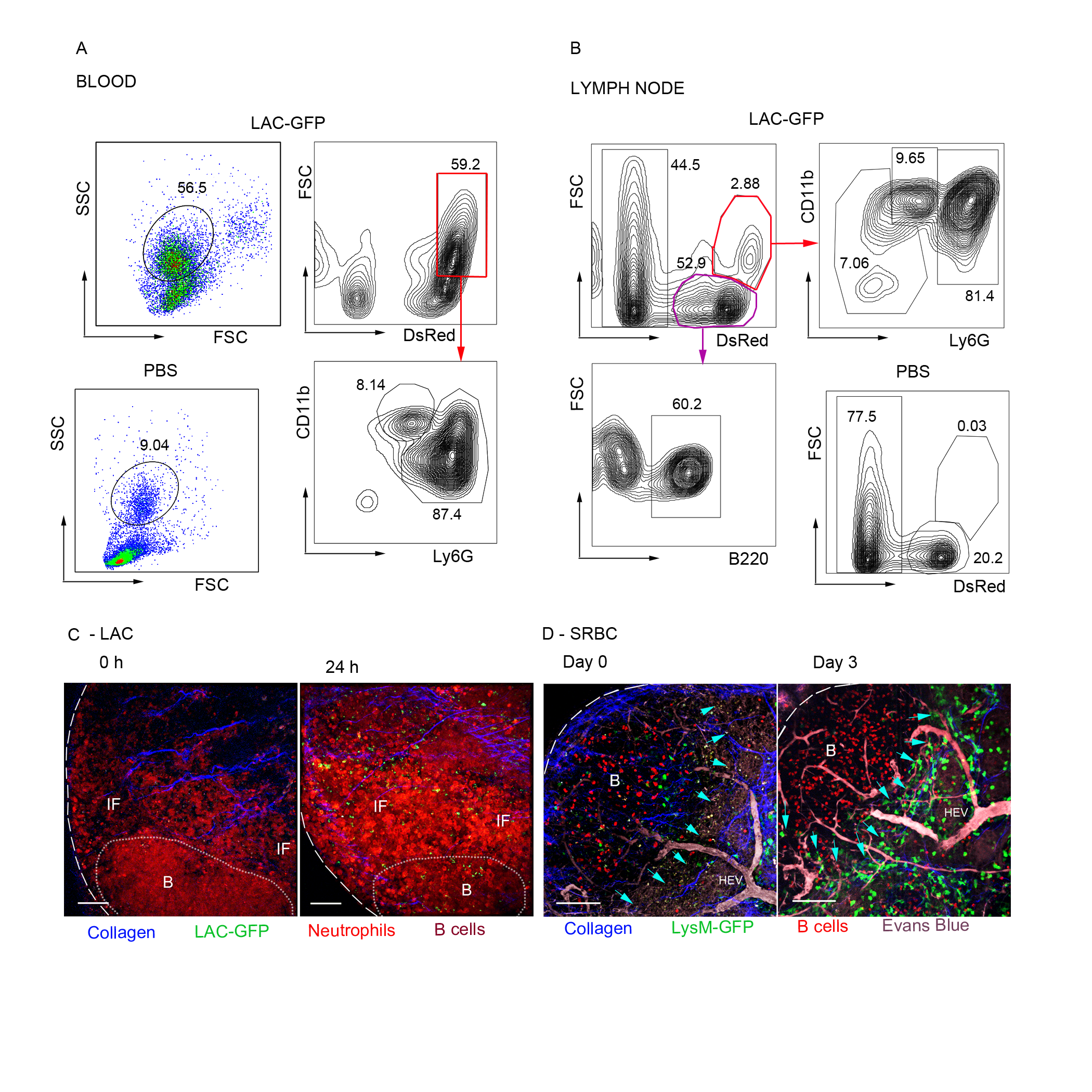

Supplement: S3 Fig — DsRed chimeric mice were injected with PBS or infected with LAC-GFP locally, near the iLN, and analyzed using flow cytometry or TP-LSM 24 h after injections. (A) Flow cytometry analysis of whole blood. Granulocyte gates in LAC-GFP infected mice (upper left) or PBS-injected control (lower left) are indicated with circles. DsRedhi population (upper right) from live cell gate and Ly6G+/CD11b+ population (lower right) from dsRedhi gate (marked with red) in LAC-GFP infected mice are shown. (B) Flow cytometry analysis of iLN cells. DsRed [hi] and [med/lo] gates in LAC-GFP infected mice (upper left) are shown. Ly6G+/CD11b+ population (upper right) from DsRedhi gate (marked with red) and B220+ population (lower left) from dsRedmed/lo gate (marked with purple) in LAC-GFP infected mice are shown. DsRed [hi] and [med/lo] gates in PBS-injected control (lower left) are shown. (A, B) Representative plots of 3 mice per group analyzed. (C) ILNs in PBS control mice (left panel) and in 24 h-infected mice (right panel) are shown. Neutrophils (dsRedhi, red). LN borders and follicular borders are shown with white dashed lines. B cell follicles (B); interfollicular zones (IFZ) are labeled. Scale bars: 50 μm. (D) LysM-GFP mice were injected with PBS or immunized near the inguinal LN with SRBC. Labeled B cells were adoptively transferred 24 h prior to imaging. ILNs in PBS control mice (left panel) and in immunized mice at day 3 after immunization (right panel) are shown. Neutrophils (GFPhi, green); B cells (CMTMR, red); blood vessels (Evans Blue, gray). LN borders are shown with white dashed lines; B cell follicles (F) and HEVs (HEV) are labeled. Scale bars: 50 μm; Z = 50 μm. (A-B) Representative images of 3 experiments. Related to Fig 3. (TIF) [file ppat.1004827.s003.tif]

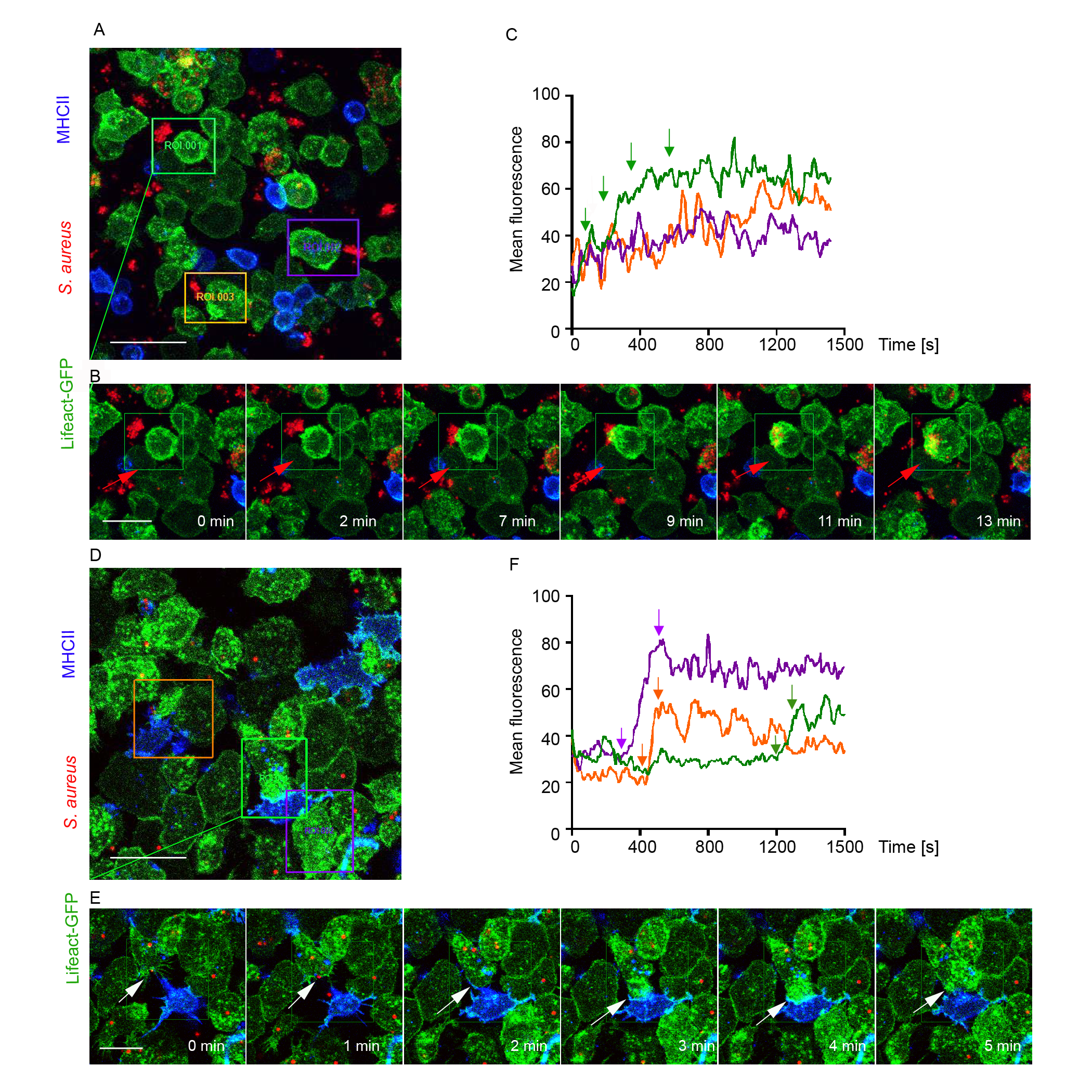

Supplement: S4 Fig — Lifeact-GFP neutrophils and B cells were co-cultured on ICAM-1+VCAM-1+KC coated surface and imaged using confocal microscopy. B cells were immunostained with anti-MHCII antibody. S. aureus bioparticles were added to the co-cultures and cells were imaged immediately (A-C) or after 2 h (D-E). (A) A confocal image of neutrophils (green) phagocytizing S. aureus (red). 3 regions of interest (cells) for quantitative analysis are indicated with squares. Scale bar: 15 μm. (B) Time-lapse series of confocal images showing steps of S. aureus uptake by a single neutrophil. F-actin clustering during the uptake is shown with red arrows. Scale bar: 7 μm; time is relative. (C) Profiles of GFP mean fluorescence in 3 cells indicated as regions of interest in (A). Changes in a curve slope shown with arrows. (D) A confocal image of neutrophils (green) interacting with B cells (blue). 3 regions of interest are indicated with squares. Scale bar: 10 μm. (E) Time-lapse series of confocal images showing steps of cell-cell contact formation between a neutrophil and a B cell. F-actin clustering during the ionteraction is shown with white arrows. Scale bar: 5 μm; time is relative. (F) Profiles of GFP mean fluorescence in 3 cells indicated as regions of interest in (D). Changes in curve slopes shown with arrows. Related to Fig 4. (TIF) [file ppat.1004827.s004.tif]

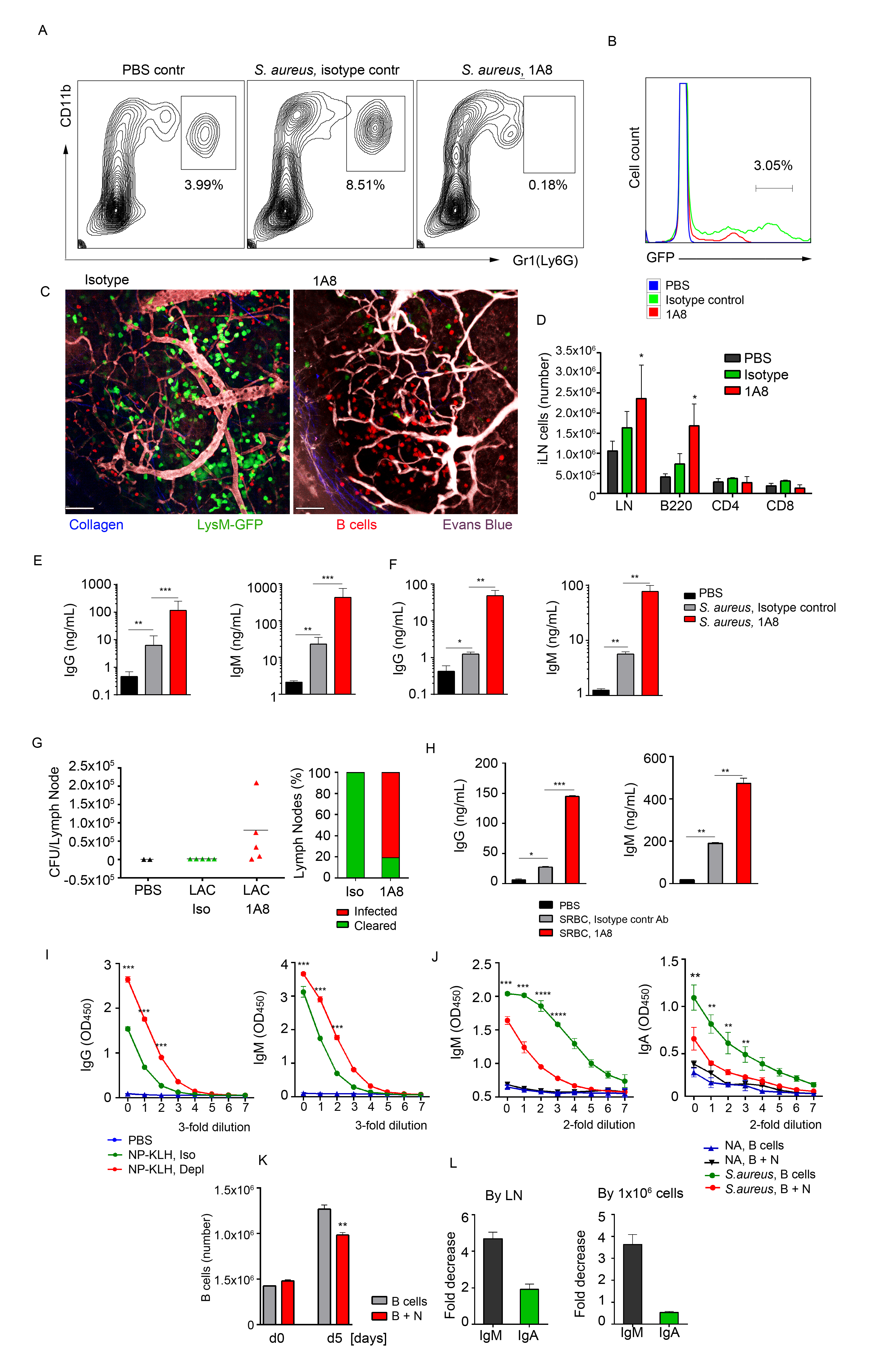

Supplement: S5 Fig — (A) Flow cytometry plots of the whole blood from PBS control or S aureus immunized mice, injected with isotype control antibody or depleting antibody clone 1A8 24 h after depletion. (B) Flow cytometry plot of iLN Ly6Ghi cell population from PBS control or S aureus immunized mice, injected with isotype control antibody or 1A8 antibody; 24 h after depletion. Data is representative of experiments using 2 mice/4 iLNs mice per group; repeated 3 times. (C) TP-LSM of iLN in neutrophil-depleted mice. Scale bars, 35 μm. N = 3. (D) Flow cytometry analysis of B and T cell populations in the iLN of neutrophil-depleted or isotype control mice 3 days after immunization. The data are shown as cell numbers. N = 4 iLNs; 3 repeats. Means ± SEM. (E) ELISA of IgG and IgM produced by B cells isolated from a single iLN from mice injected with isotype control or 1A8 antibody and immunized with S. aureus bioparticles (F) ELISA of total IgG and IgM produced by 1 x 10^5^ iLN B cells isolated from mice injected with isotype control or 1A8 antibody and immunized with S. aureus bioparticles. (E, F) B cells harvested at day 6 after immunization, cultured for 3 days. N = 4 iLNs; 3 repeats. Means ± SD. (G) CFU counts in 24 h cultures of LNs isolated from LAC-infected mice that were injected with PBS, isotype control antibody or 1A8 antibody. Number of CFU grown from one LN is shown to the left. Percent of lymph nodes that cleared LAC infection completely (0 CFU) versus those that contained LAC (CFU growth) is shown to the right. Single experiment. N = 5. (H) ELISA of total IgG and IgM produced by iLN B cells from mice injected with isotype control or 1A8 antibody and immunized with SRBC (I) ELISA of NP-KLH specific IgG and IgM produced by iLN B cells from mice injected with isotype control or 1A8 and immunized with NP-KLH. (G, H) B cells were harvested at day 7 after immunization and cultured for 3 days. N = 4 iLNs; 3 repeats. Means ± SEM. (J) B cells were isolated from LNs, and activated in vitro w [file ppat.1004827.s005.tif]

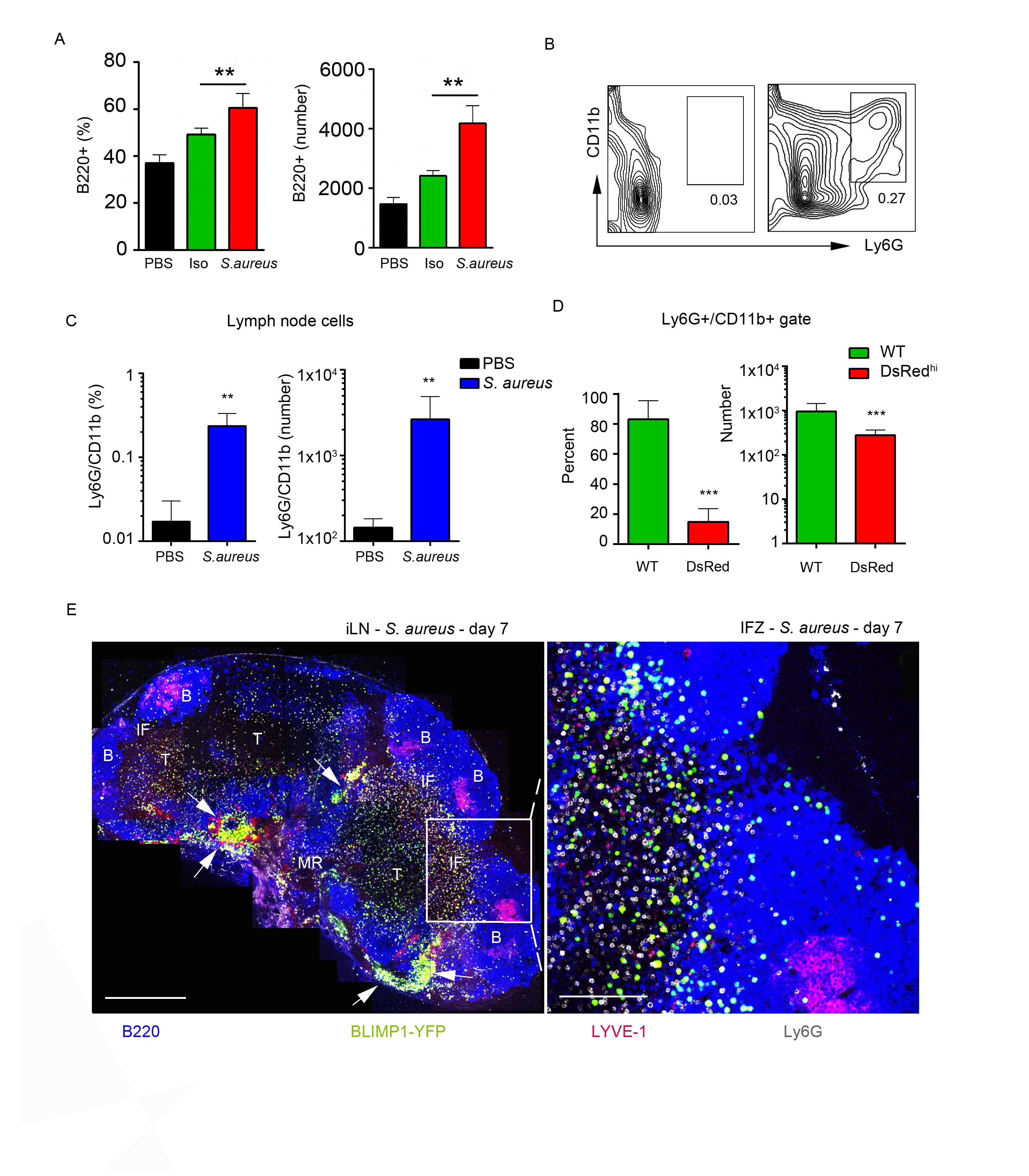

Supplement: S6 Fig — (A) Flow cytometry analysis of B220+ cell population in the iLN of neutrophil-depleted or isotype control mice 7 days after S. aureus bioparticle immunization. B220+ total cell number and percentage within the lymphocyte gate are shown. N = 4 iLNs; 3 repeats. Means ± SEM. (B-D) Flow cytometry analysis of Ly6G+/CD11b+ cell population in the iLN 7 days after immunization. (B) Representative plots of Ly6G+/CD11b+ population in PBS-injected or S. aureus immunized mice. (C) Ly6G+/CD11b+ percentage and total cell numbers within live cell gate are shown. (D) Percentages and total cell numbers of endogenous (WT) and adoptively transferred neutrophils (DsRed) are shown. Adoptive transfer was performed 12 h prior to analysis. N = 4 iLNs; 3 repeats. Means ± SEM. (E) Tiled confocal image of S. aureus immunized BLIMP1-YFP iLN at day 7 after immunization. TZ (T), IFZ (IF), medulla (MR), and LN follicles are labeled. Perivascular niches filled with BLIMP1-YFP+ cells are indicated with white arrows. Enlarged image of the IFZ (white square) is shown to the right. Immunostaining: B220 (blue), LYVE-1 (purple), Ly6G (gray). Endogenous: BLIMP1-YFP (yellow-green). Related to Fig 6. (TIF) [file ppat.1004827.s006.tif]
