# Supplementary material for: Neutrophil Recruitment to Lymph Nodes Limits Local Humoral Response to Staphylococcus aureus
Source: PLoS Pathog. 2015 Apr 17;11(4):e1004827. doi: 10.1371/journal.ppat.1004827 (PMC4401519; doi:10.1371/journal.ppat.1004827)
Supplement: S1 Table — List of targeted mouse genes with gene symbols, synonyms, full names and MGI ID numbers are provided in alphabetical order. (DOCX) [file ppat.1004827.s018.docx]

**S1 Table.**

| **Symbol** | **Synonym/Full Name** | **ID** |
| --- | --- | --- |
| B220 | CD45R, protein tyrosine phosphatase, receptor type, C | 97810 |
| BLIMP-1 | Prdm1; PR domain containing 1, with ZNF domain | 99655 |
| CXCR2 | Chemokine (C-X-C motif) receptor 2 | 105303 |
| CXCR4 | Chemokine (C-X-C motif) receptor 4 | 109563 |
| CD11c | Integrin alpha X | 96609 |
| CD11b | Integrin alpha M | 96607 |
| CD138 | Sdc1; Syndecan 1 | 1349162 |
| CD169 | Siglec-1; Sialic acid binding Ig-like lectin 1; Sialoadhesin | 99668 |
| CD38 | Cd38 antigen | 107474 |
| CD4 | CD4 antigen | 88335 |
| CD8 | CD8 antigen, alpha chain | 88346 |
| DsRed | Tg(CAG-DsRed*MST)1Nagy; transgene insertion 1, Andras Nagy | 3663358 |
| Fas | CD95; TNF receptor superfamily member 6 | 95484 |
| GL-7 | Ly77; Lymphocyte antigen 77 | 106909 |
| ICAM-1 | CD54; Intercellular adhesion molecule 1 | 96392 |
| KC | CXCL1; Chemokine (C-X-C motif) ligand 1 | 108068 |
| Lifeact-GFP | Tg(CAG-EGFP)#Rows; transgene insertion; Roland Wedlich-Soldner | 4831036 |
| Ly6G | Lymphocyte antigen 6 complex, locus G | 109440 |
| LysM | Lyz2; Lysozyme 2 | 96897 |
| LysM-GFP | Lyz2tm1.1Gra; Lysozyme 2; targeted mutation 1.1, Thomas Graf | 2654931 |
| LYVE-1 | Lymphatic vessel endothelial hyaluronan receptor 1 | 2136348 |
| MHCII | CD74 antigen; Invariant polypeptide of major histocompatibility complex, class II antigen-associated | 96534 |
| TGF-β1 | Tgfb1; Transforming Growth factor, beta 1 | 98725 |
| VCAM-1 | CD106; Vascular cell adhesion molecule 1 | 98926 |
| VE-cadherin | CD144; Cadherin 5 | 105057 |
