# Supplementary material for: Neutrophil Recruitment to Lymph Nodes Limits Local Humoral Response to Staphylococcus aureus
Source: PLoS Pathog. 2015 Apr 17;11(4):e1004827. doi: 10.1371/journal.ppat.1004827 (PMC4401519; doi:10.1371/journal.ppat.1004827)
Supplement: S1 Text — Generation of bone marrow chimeric mice is described. (DOCX) [file ppat.1004827.s019.docx]

**S1 Text. Generation of Bone Marrow chimeric mice.**

Bone marrow chimeric mice were generated in accordance with Animal Study Proposal LIR 16 entitled “Analysis of Innate Immune Function in Mice” within Comparative Medicine Branch (CMB), and has been approved by the NIAID Division of Intramural Research Animal Care and Use Committee (NIH). Shortly, C57BL/6 mice were irradiated, and then reconstituted with bone marrow (BM) from BLIMP1-YFP heterozygous or dsRed homozygous donor mice. BM prepared from euthanized donor mice was injected into the tail vein of the irradiated recipient mice. Irradiation was performed twice for a total of 1100 Rads (110 Gy) by trained animal facility personnel in compliance with radiation safety and security requirements (NIH/CMB). Donor cells were injected at 2 million cells/mouse in pharmaceutical-grade sterile PBS. Mice were recruited to experiments at least 8 weeks after BM reconstitution.
