# Supplementary material for: Neutrophil Recruitment to Lymph Nodes Limits Local Humoral Response to Staphylococcus aureus
Source: PLoS Pathog. 2015 Apr 17;11(4):e1004827. doi: 10.1371/journal.ppat.1004827 (PMC4401519; doi:10.1371/journal.ppat.1004827)
Supplement: S2 Text — Details of confocal microscopy setup and live LN sectioning are provided. (DOCX) [file ppat.1004827.s020.docx]

**S2 Text. Confocal microscopy of live iLN sections.**

Confocal imaging of live tissue sections *ex vivo* was developed as a technique for visualizing tissue architecture and cell segregation in the LNs at close to physiological conditions. Mice were euthanized using CO_2_ chamber (Braintree Scientific), and LNs were harvested and kept on ice, in 1% BSA in PBS. The LNs were trimmed from residual connective tissue and cords under Leica MZ6 modular stereomicroscope (Leica Microsystems) using surgical tweezers (Miltex). Preheated 2% agarose (Lonza) in DMEM was chilled to 40° C and immediately poured over the LNs plated in Petri dish, kept on ice. Upon agarose gel polymerization, complete lymphocyte medium was added to the Petri dishes. Agarose gel was cut into cubes each containing one LN, and sliced into 250 µm sections using [Leica VT1000 S Vibrating Blade Microtome (Leica Microsystems](http://www.leica-microsystems.com/products/total-histology/sectioning/vibrating-blade-microtomes/details/product/leica-vt1000-a/)) at speed 5, in ice-cold PBS. Tissue sections were cultured in complete lymphocyte medium in humidified incubator at 37° C for 2 h, stained with fluorescently labeled anti VE-cadherin, -LYVE1, -B220 and -Ly6G antibody (eBioscience), and washed with warm medium. Sections were held down with tissue anchors (Warner Instruments) in 14 mm Microwell Dishes (MatTek), and imaged using [Leica](http://myrtb.nih.gov/BIS/Pages/Confocal4.aspx?ResourceID=13) SP8 inverted 5 channel confocal microscope equipped with a Pecon Environmental Chamber (Pecon). Microscope configuration was set up for three-dimensional analysis (x,y,z) of cell segregation within tissue sections. Diode laser for 405 nm excitation; Argon laser for 458, 476, 488, 496, 514 nm excitation, DPSS laser for 561; and HeNe lasers for 594, 633 nm excitation wavelengths were tuned to minimal power (between 0.3–2%). Z stack of images (10–25 µm) were collected. Images were processed using Leica Application Suite (LAS, Leica Microsystems) and Imaris (Bitplane) software. Mosaic images of whole LNs were generated by acquiring multiple Z stacks using motorized stage to cover the whole LN area and assembled into a tiled image using LAS.
