# Supplementary material for: Neutrophil Recruitment to Lymph Nodes Limits Local Humoral Response to Staphylococcus aureus
Source: PLoS Pathog. 2015 Apr 17;11(4):e1004827. doi: 10.1371/journal.ppat.1004827 (PMC4401519; doi:10.1371/journal.ppat.1004827)
Supplement: S3 Text — Intravital two-photon laser scanning microscopy (TP-LSM) setup is described in detail. (DOCX) [file ppat.1004827.s021.docx]

**S3 Text. Intravital two-photon laser scanning microscopy (TP-LSM).**

TP-LSM setup included Leica SP5 inverted confocal microscope (Leica Microsystems) with dual Mai Tai lasers and 37° C incubation chamber (NIH Division of Scientific Equipment and Instrumentation Services). Additionally, the microscope was equipped with L 25.0 water-immersion objective, 0.95 NA (Olympus). Animals were anesthetized using 2% Isofluorane (Baxter), administered via nose cone mask. Skin-flip surgery was performed on an anaesthetized mouse, the mouse was placed on the cover-glass bottom stage, and the iLN was kept moisturized with warm PBS and complementarily heated with the infra-red blanket (Braintree Scientific) over the course of imaging. After imaging, animals were euthanized by cervical dislocation while still under anesthesia. Mouse calvarium BM was imaged using custom-made stainless steel stage with the head holder and the upright microscope setup. Blood vessels were visualized via intravenous injection of 0.5 % Evans Blue dye (Sigma Aldrich) in sterile PBS at 1 ml/kg; or Q-dot 665 (Molecular Probes, Invitrogen), diluted in PBS according to the manufacturer’s protocol. For time-lapse imaging, Z stack consisting of 5–10 single planes (2–5 μm each over a total tissue depth of 10–50 μm) was acquired every 9–15 seconds for a total observation time between 1 to 6 hours. Post-acquisition image processing was performed using Leica Application Suite (Leica Microsystems), Imaris (Bitplane), and Huygenes (SVI) software. The intensity of fluorescence in Lifeact-GFP cells was analyzed using ImageJ (National Institutes of Health). The curves of Lifeact-GFP fluorescence intensity were built and statistical analysis performed using GraphPad Prism. Cell migration parameters were evaluated using Imaris and GraphPad Prism.
